# Unraveling flavor formation mechanism of cigar smoke through volatile compounds in cigar smoke and potential precursors in cigar tobacco

**DOI:** 10.3389/fpls.2025.1672119

**Published:** 2025-09-25

**Authors:** Xiaowei Zhang, Tianyu Dong, Dan Chen, Haowei Sun, Anchuan Xu, Chunqiong Wang, Dan Li, Jieyun Cai, Lingduo Bu, Ke Zhang, Haitao Chen

**Affiliations:** ^1^ Yunnan Tobacco Quality Inspection & Supervision Station, Kunming, Yunnan, China; ^2^ Beijing Key Laboratory of Flavor Chemistry, Beijing Technology and Business University, Beijing, China; ^3^ Technology Center, China Tobacco Yunnan Industrial Co., Ltd., Kunming, Yunnan, China; ^4^ Yunnan Tobacco Company, Yuxi, Yunnan, China

**Keywords:** cigar smoke, solvent-assisted flavor evaporation (SAFE), comprehensive two-dimensional gas chromatography with time of flight mass spectrometry (GC×GC-TOFMS), flavor precursor, relative odor activity value (ROAV), orthogonal partial least squares discriminant analysis (OPLS-DA), flavor formation mechanism

## Abstract

The flavor of cigar smoke directly influences the consumer’s willingness to purchase. However, the analysis of cigar smoke was lack. In this research, a total of 235 volatile compounds were identified by GC×GC-TOFMS in cigar smoke from Yuxi, Dehong and Pu’er regions of Yunnan, including 72 alkenes, 47 aromatic hydrocarbons, 42 heterocyclics, 28 ketones, 8 alcohols, 4 aldehydes, 3 esters, 13 phenols, 5 acetonitrile and 13 others. ROAV analysis indicated that *o*-cresol, guaiacol, 2,6-dimethyl-phenol, 4-hydroxy-3-methoxystyrene, *(E,E)*-2,4-hexadienal, butylbenzene, naphthalene, nicotine and D-limonene might be the key flavor compounds. By OPLS-DA, 2-methyl-pyridine, 2,4-dimethylfuran, 2-carene, 13-heptadecyn-1-ol, 1-ethyl-2-methyl-benzene, 2,3,5,8-tetramethyl-1,5,9-decatrien, 1-ethyl-4-methyl-benzene, *β*-elemene, methyl isobutyl ketone and 3-methylcyclopentanone were found to be the differential volatiles distinguishing samples from different areas. Eventually, 5 groups of potential flavor precursors were summarized and identified (cembranoid, phenylalanine, chlorophyll, carotenoid and reducing sugar). This research has provided theoretical guidance for the improvement of flavor quality of cigar smoke in Yunnan.

## 1 Introduction

Cigar is a tobacco product, rolled from dried and fermented tobacco. Cigar is divided structurally into 3 sections from the inside out: the filler, binder and wrapper. The wrapper, which is the smallest part of the quality, is particularly important for cigars, since it contributes to the quality and appearance of the cigar (Peng et al., 2025). Cigar hold a growing market share in the tobacco industry due to its rich smoke and unique flavor (Ji et al., 2024). For example, cigar sales in the United States have gradually increased compared to cigarette (Jensen et al., 2025). According to the statistics of China Research Puhua Industry Research Institute, the compound growth rate of Chinese cigar consumption has been as high as 18.9% in the past few years. There is no doubt that emerging markets, such as China, are becoming important drivers for the cigar industry. It is well known that the growing environment, has a significant effect on the quality of cigar tobacco, such as light (Peng et al., 2025), temperature (Ren et al., 2023), rainfall (Ahmed et al., 2025) and soil (Shen et al., 2024). Therefore, the major global production regions for cigar tobacco include Cuba, Brazil, Dominica, Indonesia, China and others (Zheng et al., 2022). In China, the geographical environment of the Yunnan region is more suitable for the growth of cigar tobacco, which has a richer flavor and better quality. Yunnan’s tobacco production accounts for about 40% of Chinese total (Tie et al., 2024). In Yunnan, cigar tobacco cultivation is mainly distributed in Dehong, Yuxi, Lincang, Pu’er, etc (Song et al., 2022).

In the Web of Science Core Collection, the literature on cigars represents only about 1% of tobacco research. At present, cigar research has focused on smokers, age, health, and risk. However, there were less studies on cigar flavor and even fewer on cigar smoke. It is worth noting that research on the flavor of cigar has mainly focused on the evaluation of flavor differences between different regions or stages of fermentation. The characteristic aroma attributes of cigars include tobacco, creaminess, cocoa, leather, baking, herbaceous, leathery, woodsy, and fruity notes (Zhou et al., 2024a). It has been shown that the main volatile flavor compounds in cigar tobacco were aldehydes and ketones, and the flavor of cigar tobacco varied significantly from region to region, which was related to the microbial community (Zheng et al., 2022). Zhou et al. have found 24 compounds (2-heptanone, n-butanol, 2,6-dimethylpyrazine, 2-furfuryl methyl sulfide etc.) to be the key differential components of 6 Chinese cigars by GC-IMS combined with PLS-DA (Zhou et al., 2024a). 12 volatile organic compounds, including (S)-(-)-nicotine, neophytadiene, *α*-tolualdehyde, npentanal, benzaldehyde-M and so on, were identified as differential markers in the fermentation process of cigar tobacco leaves by PLS-DA and VIP value (Zhang et al., 2024). For cigar tobacco, fewer systematic studies have been conducted on its key flavor compounds.

Tobacco has a highly complex chemical composition and tobacco is smoked during the burning process. As a consequence of that, it is difficult to completely characterize the overall quality of tobacco by only analyzing it in its non-combustible state (cigar tobacco leaves). In this view, the flavor analysis of smoke during cigar burning is highly significant for the evaluation and enhancement of the quality of cigars. Yang et al. analyzed representative Chinese cigars and Cuban cigars by GC-MS combined with chemometrics, and the results showed that the Cuban cigars contained a moderate level of nicotine content, and higher contents of phytol, neophytadiene, 3-methylpentanoic acid, and (+)-*δ*-cadinene (Yang et al., 2024). However, there has been a gap in the analysis of volatile flavor compounds in cigar smoke from Yunnan, an important production area for cigar tobacco in China. Therefore, it is essential to explore the flavor code of Yunnan cigar smoke.

The comprehensive two-dimensional gas chromatography with time of flight mass spectrometry (GC×GC-TOFMS) has higher peak capacity and higher sensitivity than GC-MS, and can separate and recognize more volatile organic compounds (VOCs) in the samples. Therefore, it is suitable for analyzing volatile compounds in complex matrixes and is able to identify more trace compounds efficiently. Currently, it has been widely used in tobacco chemistry (Schwanz et al., 2019), alcohol chemistry (He and Jeleń, 2025), tea chemistry (Wang et al., 2024) and so on. In summary, GC×GC-TOFMS is appropriate for the analysis of VOCs in the complex cigar smoke.

The smoke produced by cigar tobacco after combustion is the product of complex chemical reactions. There is no denying that the formation of cigar smoke aroma compounds requires processes such as pyrolysis, oxidation and condensation of tobacco precursors. Based on the precursors of the volatile compounds, the volatile compounds in the cigar might include cembrenediol degradation products (Wang et al., 2020), carotenoid degradation products (Zhang et al., 2017), phenylalanine conversion products (Gao et al., 2019), chlorophyll degradation products (Gutbrod et al., 2021), and Maillard reaction products (Zhou et al., 2024b). However, the sources of the dominant flavor compounds in cigar smoke require more in-depth research and analysis to determine.

In this study, we obtained smoke samples from three different cigar tobaccos originating from Yunnan using a single-orifice smoker and a smoke collection system. Secondly, solvent-assisted flavor evaporation (SAFE) was used to extract VOCs from cigar smoke. Then, GC×GC-TOFMS was used to characterize the VOCs in the three smoke samples. At the same time, relative odor activity values (ROAVs) were calculated in order to Explore the relative aroma contribution of each component. Last but not least, we determined the levels of potential precursors of cigar smoke in tobacco leaves. correlation analysis methods were used to preliminarily identify the formation pathways of major flavor compounds in cigar smoke. Based on the content of potential precursors and literature, we have identified the sources of the main flavor compounds in cigar smoke.

## 2 Materials and methods

### 2.1 Materials

**Figure 1:**
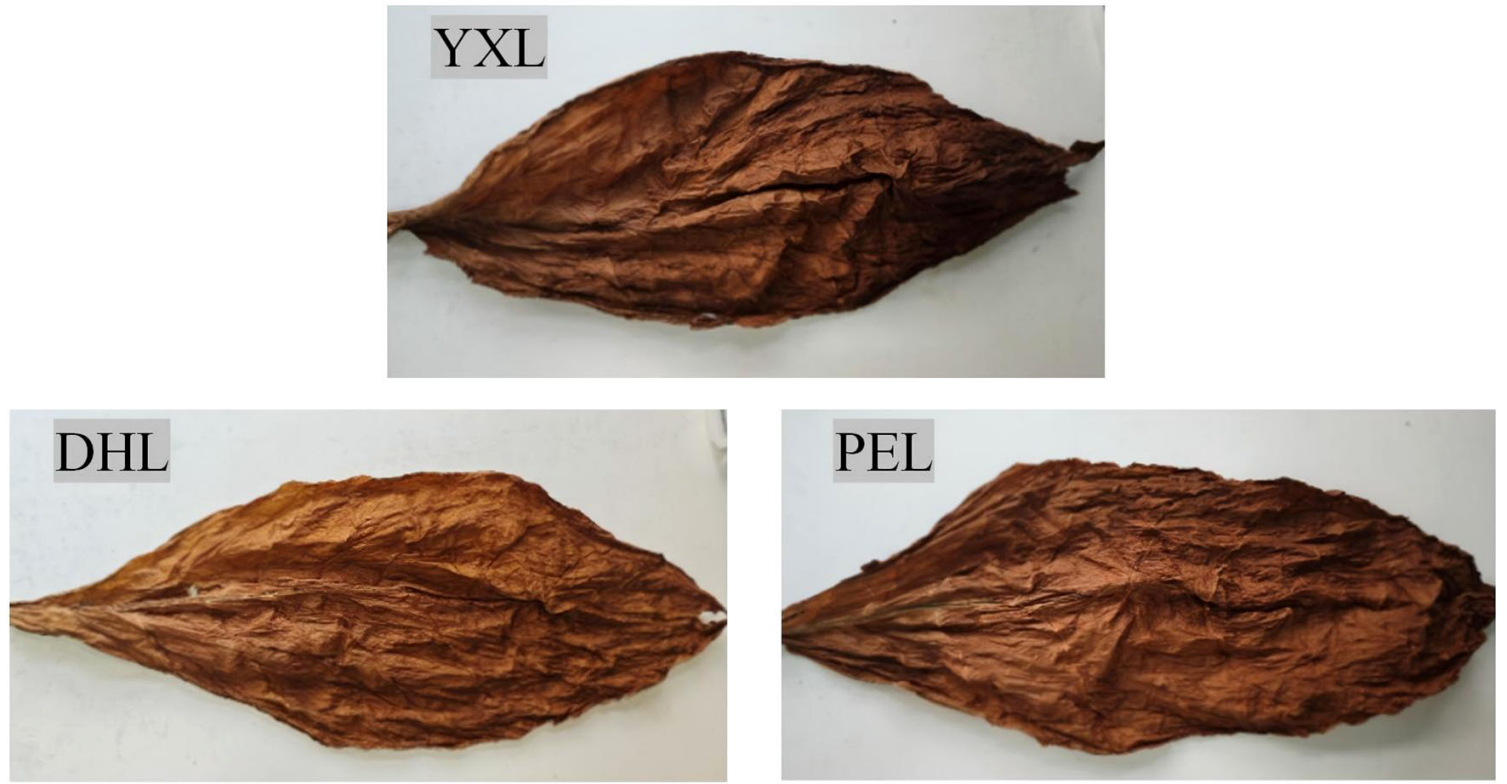
Cigar leaves samples from 3 regions in Yunnan (Yuxi, Yunnan, YXL; Dehong, Yunnan, DHL; Pu’er, Yunnan, PEL).

The cigar tobacco samples were provided by Yunnan Provincial Tobacco Quality Supervision and Inspection Station. Sample 1: Yunxue No. 6 central cigar core tobacco from Yuxi City, Yunnan Province (YXL); Sample 2: Yunxue No. 36 central cigar-coated tobacco from Mangshi, Dehong Dai-Jingpo Autonomous Prefecture, Yunnan Province (DHL); Sample 3: Yunxue No. 2 central cigar-coated tobacco from Pu’er City, Yunnan Province (PEL). The above samples were shown in the form of tobacco leaves in **Figure 1**. In addition, the samples had the same conditions of grade, cultivation, drying and fermentation technology. All samples were equilibrated in a constant temperature and humidity box (temperature 20°C, humidity 70%-75%) for 48 h and stored for reserve.

### 2.2 Chemicals

Dichloromethane and sodium sulfate were purchased from Mreda (Beijing, China). Liquid nitrogen, high purity helium (≥ 99.999%) and high purity nitrogen (≥ 99.999%) were purchased from Beijing Ruizhi Hanxing Technology Co., Ltd (Beijing, China). 2-Octanol (99%), *β*-Carotene (≥96%) and *β*-Cembrenediol (≥98%) were purchased from Aladdin (Shanghai, China). n-Alkanes (C_7_–C_35_) were purchased from Sigma-Aldrich (Shanghai, China). Phenylalanine and 2,7,11-cembratriene-4,6-diol (*α*-Cembrenediol) were purchased from Yuanye (Shanghai, China). Chlorophyll A was purchased from Macklin (Shanghai, China). Mix-5 in Acetonitrile: Water=1:3 was purchased from TMstandard (Changzhou, Jiangsu, China). Bis(trimethylsilyl)trifluoroacetamide (GR), 5-sulfosalicylic acid dihydrate (GR), 2,4-dinitrofluorobenzene (GR), butylated hydroxytoluene (GR) and acetonitrile (HPLC) were purchased from Anpel (Shanghai, China). N,N-Dimethylformamide (AR), sodium bicarbonate (AR), potassium phosphate monobasic (AR), petroleum ether (AR), hydrochloric acid (AR), sodium hydroxide (AR) and potassium sodium tartrate (AR) were purchased from Damao (Tianjin, China). Copper (II) sulfate (AR), sulfuric acid (AR) and ferric sulfate (AR) were purchased from Guangzhou Chemical Reagent Factory (Guangzhou, Guangdong, China).

### 2.3 Rolling of single-filler cigars

Firstly, the whole cigar tobacco leaf was sprayed with water to dampen it and treated to reach a level of softness that would allow it to be rolled. Next, single-feed cigar cigarillos were hand-rolled using a cigar-rolling mold (Cigar shaper (secret 132), cigar cutter and cigar binder (Wuhou Jinggong advertising design service department, Chengdu, Sichuan, China)). The cigar-rolling samples (47 mm circumference × 110 mm length, 6.0 ± 0.2 g) were exposed to a cool, ventilated place for 3 days. After that, they were equilibrated at (20 ± 2) °C and (70 ± 2) % relative humidity for 72 h. Three cigar cigarette samples were prepared repeatedly for three times.

### 2.4 Collection of cigar smoke

The first step was the preparation of the cigar single channel smoking machine (CSM 1000) (Hefei Institutes of Physical Science, Chinese Academy of Sciences, Anhui, China). Cambridge filter (55 mm) was placed into the smoke trap, with the rough surface facing the air inlet direction, and check whether it was properly assembled. Then, the Compressed air filter XF7-16 (Campbell Hausfeld, Illinois, USA) was turned on to make the air pressure ≥ 0.5 MPa, and the preheating procedure of the smoking machine was started for 30 min. After that, the air leakage test was conducted to ensure the integrity of the gas path of the trap. Finally, the accuracy of the suction capacity was tested, and the suction volume parameters were calibrated by the displacement of the soap film in the soap film flowmeter.

The single material cigar sample was smoked through the cigar single channel smoking machine to obtain cigar smoke. The parameters during the smoking process were set according to CI international standards (suction capacity of 55 mL, suction frequency of 30 s, suction duration of 2 s, fixed number of 50 puffs). On the one hand, particulate matter in mainstream smoke would be intercepted by Cambridge filters. But on the other hand, a continuous capture system was required to be constructed for volatile components in cigar smoke. This capture system consisted of three solvent absorption bottles connected in series, with 80 mL of dichloromethane, respectively. The adjusted cigar was inserted into the hole of the trap’s clamping device. In particular, we would ensure that the end of the butt was in contact with the perforated cushioning spacer and adjust the detector scale to 28 mm of the tobacco stick. After completing the above, started the ignition program to begin cigar smoking. After smoking, a 2-puffs blank was performed to remove residual components from the orifice, the butt was removed, and the smoking operation was concluded.

### 2.5 Isolation of volatiles by SAFE

Three bottles of extraction solution were mixed and Cambridge filter was put into the extraction solution for 1 h. 2-Octanol (80 *μ*L, 1.26 g/L) was added to the solution as an internal standard. The extraction solution was introduced into the SAFE system and the phase separation of volatile compounds was accomplished in an ultra-high vacuum environment (10–^5^ mbar). The resulting fraction was then dehydrated by anhydrous sodium sulfate after returning to room temperature. After dehydration, the solution was further concentrated to 1.5 mL by Vigreux fractionation system (50 cm × 1 cm). Finally, the concentration was filtered through a microporous membrane and concentrated to 1 mL by nitrogen purging technique. All experiments were repeated for 3 times. Eventually, three cigar smoke samples were obtained. They were Yuxi cigar tobacco smoke (YX), Dehong cigar tobacco smoke (DH) and Pu’er cigar tobacco smoke (PE). The samples were sealed and stored in an ultra-low temperature refrigerator (- 40°C) for further analysis.

### 2.6 GC×GC-TOFMS analysis

An Agilent 8890B GC (Agilent Technologies, Palo Alto, CA, USA) system equipped with a split/splitless injector and a solid-state thermal modulator SSM1810 with SV Series Modulator (C7-C40) (Xuejing Electronic Technology Co., Ltd., Shanghai, China) coupled with an Agilent 7250A TOFMS detector (Agilent Technologies, Palo Alto, CA, USA) was used for analysis. Helium (99.999%) was used as the carrier gas and delivered at a fixed flow rate of 1.3 mL/min to the column. The inlet temperature of GC was 250 °C, injection volume 1 *μ*L, split ratio 20:1. The chromatographic columns were a DB-5MS (60 m×0.25 mm×0.25 *μ*m) in the first dimension (1D) and DB-17MS (0.85 m×0.25 mm×0.15 *μ*m) in the second dimension (2D). For the DB-5MS column, the oven temperature was initially 50 °C, followed by a 5 min hold; increased to 150 °C at a rate of 1 °C/min; and finally increased to 240 °C at a rate of 2 °C/min, followed by a 10 min hold. The modulation period was 5 s; inlet temperature, 0 °C (relative to GC column box temperature); outlet temperature, +30 °C (relative to GC column box temperature); cold zone temperature, -51 °C. The TOF detector condition: ionization energy, 70 eV; mass range, 40–650 amu; Scanning rate, 50 spectrum/s; transfer line temperature, 280 °C; ion source temperature, 210 °C; and solvent delay, 4.5 min.

### 2.7 Qualitative and quantitative analysis

The mass spectrometry data were automatically peak detected and merged by Canvas 2.0 (Xuejing Electronic Technology Co., Ltd., Shanghai, China), with a detection threshold set for all peaks greater than a signal-to-noise ratio of 20. After removing the invalid peaks such as column loss, the peaks were matched according to the NIST 17 library. Then, the volatile compounds in the samples were identified by retention index (RI). The quantitative analysis was performed by the internal standard method, i.e. the relative content of each volatile compound in cigar smoke samples was quantified by the ratio of the peak area of the component to be measured in the sample to the peak area of the internal standard.

### 2.8 Determination of potential precursors of cigar smoke in cigar tobacco leaves

The samples were homogenized by homogenizer or pulverized by high-speed pulverizer, and then stored in sample bottles.

#### 2.8.1 Determination of cembrenediols by GC-MS

0.5 g of sample was added to a 100 mL conical flask. Next, 50 mL of dichloromethane extraction solution was accurately added to the conical flask and ultrasonically shaken for 10 min. In particular, the extract was filtered using filter paper containing 5 g of anhydrous sodium sulfate. 10 mL of the filtrate was concentrated at 40°C to remove solvent. 1000 *μ*L of derivatization reagent was used to rinse the residual sample, and the resulting solution was transferred to a chromatographic vial. The derivatization reaction was carried out in a water bath at 75 °C for 60 min under airtight conditions to obtain the sample solution for GC-MS analysis.

GC-MS analysis was performed by an Agilent Gas Chromatography-Mass Spectrometer 7890-5977B (Agilent Technologies, Palo Alto, CA, USA). Separation was performed using HP-5MS column (30 m×0.25 mm i.d., 0.25 *μ*m). Helium was used as the carrier gas and delivered at a fixed flow rate of 1 mL/min to the column. For the HP-5MS column, the oven temperature was initially 150 °C; increased to 215 °C at a rate of 20 °C/min; and finally increased to 290 °C at a rate of 10 °C/min, followed by a 20 min hold; injection volume 1 *μ*L, split ratio 10:1. The mass detector condition: ionization energy, 70 eV; Ion source temperature, 230 °C, Quadrupole temperature 150 °C; scanning mode, SIM; mass range, m/z 40-350; and solvent delay, 5 min; *α*-2,7,11-cedratriene-4,6-diol derivatives: quantitative ion 169 m/z, qualitative ions 143, 211 m/z; *β*-2,7,11-cedratriene-4,6-diol derivatives: quantitative ion 169 m/z, qualitative ions 143, 211 m/z.

#### 2.8.2 Determination of phenylalanine by HPLC-PDA

Placed the sample (5 g) in a 50 mL centrifuge tube, added water (5 mL) and mix well. Then, sonicated it for 10 min, boiled for 15 min, and cooled. Thereafter, sulfosalicylic acid solution (35 g/L, 15 mL) was added, vortexed and centrifuged (10500 r/min, 20 min). All the supernatant was transferred to a volumetric flask (25 mL), fixed with 35 g/L sulfosalicylic acid solution, and set aside (mixed standard stock solution, MSSS).

200 *μ*L of MSSS or 1 mL of sample solution was placed in a centrifuge tube (15 mL), and 0.5 mol/L sodium bicarbonate solution (pH=9.0) 1 mL and 1% DNFB derivatization solution (2,4-dinitrofluorobenzene, abbreviated as DNFB, 1 mL DNFB, acetonitrile to 100 mL) 1 mL, was mixed by vortexing, and then put in a water bath at 60 °C for 10 min, and then cooled. The solution was fixed to 5 mL with 0.01 mol/L KH2PO4 solution (pH 7.0), mixed well, and kept aside for subsequent determination and analysis.

A Shimadzu LC-40 high performance liquid chromatography (HPLC) (Shimadzu, Kyoto, Japan) connected to a photo-diode array (PDA) was used for PHE determination. Separation was performed using an waters symmetry C18 chromatographic column (5 *μ*m*250 mm*4.6 mm), with a 25 min gradient program of 0.05 mol/L aqueous solution of CH3COONa containing 1% DMF (pH=6.5) (A) and acetonitrile (B): 16% B (1 min), 16–31% B (4 min), 31–36% B (9.5 min), 36–55% B (11 min), 55–60% B (12 min), 60–100% B (15 min), 100% B (20 min), 100–16% B (25 min). The HPLC conditions were as follows: column temperature, 35 °C; injection volume, 10 *μ*L; PDA detection 360 nm; flow rate, 1.5 mL/min.

#### 2.8.3 Determination of chlorophyll by HPLC-PDA

The sample (5 g) was weighed into a centrifuge tube (50 mL). Then, 15 mL of 0.1% BHT-methanol solution (butylated hydroxytoluene, abbreviated as BHT) was added, vortexed and mixed, extracted by ultrasonication (2 min), and centrifuged (8000 r/min, 5 min). Thereafter, the upper solution was transferred to a 150 mL rotary evaporation flask, the extraction was repeated three times and the extracts were combined in the evaporation flask. The extract was concentrated by rotary evaporation (40 °C) and nitrogen blowing. Then, the components in the distillation flask were washed out with 0.1% BHT methanol solution, transferred to a volumetric flask (5 mL), and fixed with 0.1% BHT methanol solution. The solution was passed through 0.45 *μ*m organic system microporous filter membrane, and then the content of chlorophyll was determined by HPLC.

A Shimadzu LC-40 high performance liquid chromatography (HPLC) (Shimadzu, Kyoto, Japan) connected to a photo-diode array (PDA) was used for chlorophyll determination. Separation was performed using a waters symmetry C18 chromatographic column (5 *μ*m*250 mm*4.6 mm). The HPLC conditions were as follows: mobile phase, methanol (100%); column temperature, 30 °C; injection volume, 10 *μ*L; PDA detection 665 nm; flow rate, 1.0 mL/min.

#### 2.8.4 Determination of carotene by HPLC-PDA

The sample (0.5 g) was added to a centrifuge tube (50 mL), 30 mL of petroleum ether was added, shaken gently, ventilated, capped, and shaken for 10 min at room temperature, and the supernatant was separated. The sample was transferred into a centrifuge tube for the second extraction as described above. The organic phases were combined and washed to near neutrality with water. The aqueous phase was removed and the organic phase was dehydrated by anhydrous Na_2_SO_4_. The filtrate was transferred to a 250 mL evaporation flask and concentrated under reduced pressure in a rotary evaporator (40 °C ± 2 °C). The concentrate was blown dry with nitrogen, followed by accurate addition of dichloromethane (1.0 mL) using a pipette, capping the bottle in order to fully dissolve the extract.

#### 2.8.5 Determination of reducing sugar by titration

K_2_MnO_4_ titration method was used to determine reducing sugar (GB5009.7–2016 the second method).

### 2.9 Statistical analysis

Experimental data were collected and organized using Excel (Microsoft Office 2021, Redmond, WA). The results of the experiments were expressed as the mean of three experiments ± standard deviation. Heat map was created by TBtools software (https://github.com/CJ-Chen/TBtools). Bar charts and venn diagram were plotted using Origin version 2025b (OriginLab Corporation, Northampton, MA.). Orthogonal partial least squares discriminant analysis (OPLS-DA) analysis was conducted using Simca 14.1 (Umetrics, Sweden).

## 3 Results and discussion

### 3.1 Analysis of VOCs in cigar smoke

**Figure 2:**
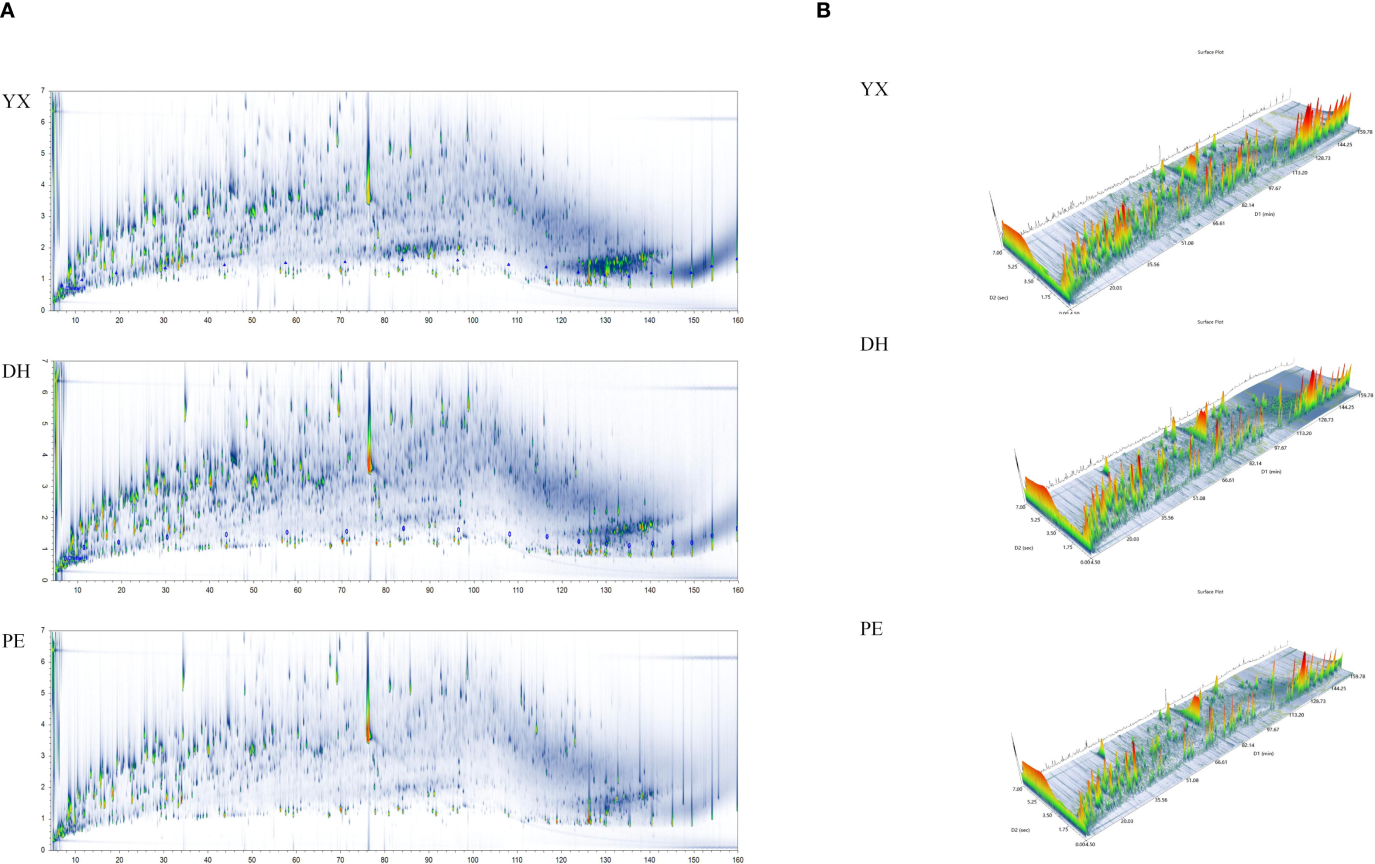
The results of GC×GC-TOFMS of cigar smoke samples from 3 regions in Yunnan (Yuxi, Yunnan, YX; Dehong, Yunnan, DH; Pu’er, Yunnan, PE). **(A)** The full two-dimensional chromatographic profiles (TIC) of cigar smoke samples **(B)** The 3D view of the full 2D chromatograms of cigar smoke samples.

In this study, GC×GC-TOFMS was used to analyze volatile compounds in cigar smoke from different regions of Yunnan (Yuxi, Dehong, Pu’er). **Figure 2** summarized key findings from GC×GC-TOFMS analyses, illustrating the complexity and diversity of volatile compounds in different cigar smoking. Of these, **Figure 2A** showed the full two-dimensional chromatographic profiles (TIC) of the three samples. **Figure 2B** exhibited a 3D view of the full 2D chromatograms of three cigar smoke samples. From the above graphs, it can be visually summarized that cigar smoke samples had a complex composition of flavor compounds. In particular, there were significant differences in the flavor compounds of cigar smoke from different regions.

**Figure 3:**
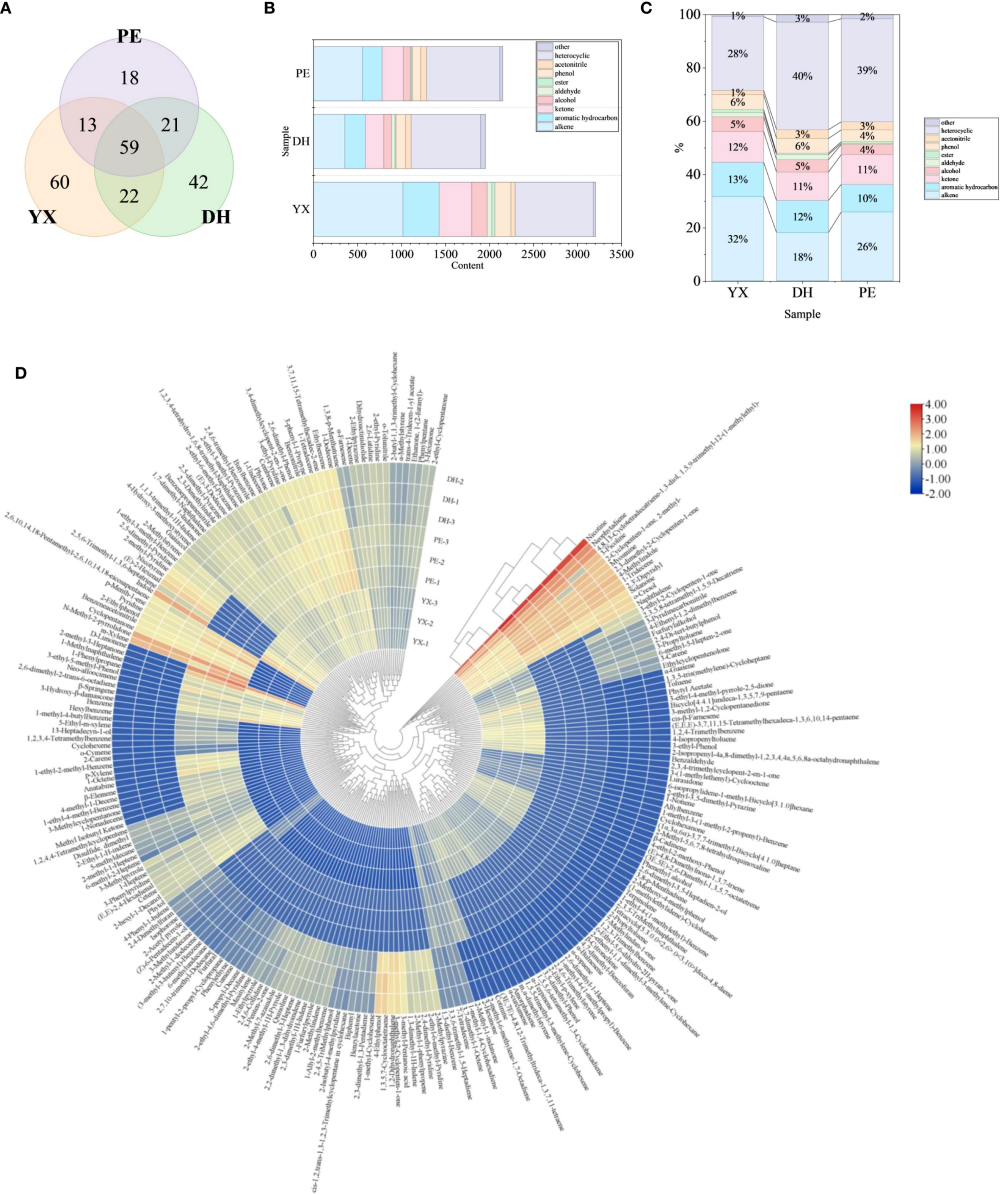
Volatile flavor compounds in cigar smoke samples. **(A)** Analyzing the similarities and differences of cigar smoke from 3 regions by Venn diagram. **(B)** The content of various kinds of volatile compounds in 3 cigar smoke samples. **(C)** Proportion of each kind of volatile compound. **(D)** The content of volatile compounds in 3 cigar smoke samples.

After matching and screening with NIST 17 and RI characterization, a total of 235 volatile compounds were identified in the three samples, including 72 alkenes, 47 aromatic hydrocarbons, 42 heterocyclics, 28 ketones, 8 alcohols, 4 aldehydes, 3 esters, 13 phenols, 5 acetonitrile and 13 others (**Supplementary Table S1**). Especially, cigar smoke was rich in heterocyclic compounds, mainly pyridines (20), pyrroles (7), pyrazines (6), indoles (4), furans (3), quinoline (1) and quinoxaline (1). In order to present the similarities and differences of cigar smoke in different geographical areas more clearly, Venn plot (**Figure 3A**) has been drawn. A total of 59 volatile compounds were shared by the cigar smoke from the three regions. In addition, there were 60, 42, and 18 volatile compounds that were unique to YX, DH, and PE, respectively. It is noteworthy that YX has the highest number of volatile compounds (154) and PE has the lowest (111). Above, volatile compounds from three types of cigar smoke were analyzed from the view of the types of compounds. However, there is no doubt that the content of each compound was also an important factor for revealing the flavor code of cigar smoke.

From the content of volatile compounds, YX had the most abundant volatile components among the 3 samples (3204.12 *μ*g/L) (**Figure 3B**). Next came to PE (2148.99 *μ*g/L) and DH (1953.63 *μ*g/L), with their volatile component levels being relatively similar. In all the 3 samples of cigar smoke, the presence of heterocyclic compounds was abundant (40% in DH, 39% in PE, 28% in YX) (**Figure 3C**). It is noteworthy that the kinds and content of volatile compounds in YX were significantly higher than the other two samples. This might be attributed to its geographical locations, where the growing environment might be more beneficial for the formation of volatile compounds. Research has shown that organic heterocyclic compounds are particularly abundant in cigar. Heterocyclic compounds such as pyrroles and furans might be associated with the Maillard reaction, which might provide tobacco toasting and nutty aroma to cigars during combustion (Chen et al., 2025; Zhu et al., 2025). In particular, 2-pentylfuran and trimethylpyrazine have been identified as typical key flavor substances in cigar tobaccos (Chen et al., 2025). However, these 2 major volatile compounds were not detected in this research, which may result from differences in sample state, origin, or differences in detection methods. Especially, the nicotine content had the highest percentage in all three cigar smoke samples. Nicotine also showed the highest detection intensity among other detection methods such as microwave plasma torch mass spectrometry (Tang et al., 2024). In addition, the content of alkene in cigar smoke was also high (32% in YX, 26% in PE, 18% in DH). Alkenes are widely found in nature. Moreover, terpenes play prominent roles in the formation of various plant flavors. In YX and PE, the most abundant terpene was limonene, but in DH, the most abundant terpene was neophytadiene. These two VOCs may have a special contribution to the formation of cigar smoke flavor. The differences between terpene compositions might have caused the flavor differences among the three regional samples, which led to consumer choice (Marć et al., 2024). Next, the proportion of aromatic hydrocarbon and ketone compounds in the three types of cigar smoke was similar, accounting for approximately 10% of the total volatile compounds, respectively. In YX, the most abundant aromatic hydrocarbon was benzene, which was not present in DH. In DH and PE, the most abundant aromatic hydrocarbon was m-xylene, while m-xylene was not present in YX. In the three samples, the most abundant ketones were solanone (YX), 2,3-dimethyl-2-cyclopenten-1-one (DH) and 2-methyl-3-heptanone (PE). Thus, these differences might directly contribute to flavor differences. Subsequently, the proportion of phenols and alcohols in the three samples was also similar, accounting for approximately 5% of the total volatile compounds, respectively. Lastly, cigar smoke also comprised trace quantities of nitriles, esters and aldehydes.

In order to present the similarities and differences in the volatile compound compositions of the three samples more visually, a heat map has been plotted (**Figure 3D**). Cluster analysis showed that the differences between YX and DH were more remarkable, with PE falling in between. The gradation of colors from red to blue in the heat map. The 2 most highly present volatile compounds in all three cigar smoke samples were nicotine and neophytadiene. There is no doubt that nicotine is the most important volatile compound in the smoke from tobacco. It has been shown that nicotine and neophytadiene were prominent in cigars, accounting for about 90% of the total VOCs (Wu et al., 2024). It has been in general agreement with the findings of this paper, but its percentage was not so high. This might be due to the difference in the detection methods, employing GC×GC-TOFMS, which greatly improves the detection level of other VOCs. It is noteworthy that only about 4% of the compounds, which exhibited a reddish hue in all three samples, were relatively high in cigar smoke, so these compounds may play a more prominent role in the flavor formation of cigar smoke. At the same time, most of the compounds showed a blue hue in the heat map and were present at low levels in the cigar smoke samples, in the range of 0-30 *μ*g/L. In addition, 2-methylstyrene, 1-ethyl-2-methyl-benzene, 2,5-dimethyl-pyridine, 2-methyl-pyridine, nicotyrine, *(E)*-2-hexenal, 2,5,6-trimethyl-1,3,6-heptatriene, indole, 2,6,10,14,18-pentamethyl-2,6,10,14,18-eicosapentaene, *p*-menth-1-ene, pyridine, 2-ethylphenol, benzeneacetonitrile, cyclopentanone, N-methyl-2-pyrrolidone, *m*-xylene, D-limonene, 2-methyl-3-heptanone, 1-methylnaphthalene, 1-phenylpropane, 3-ethyl-5-methyl-phenol, neo-alloocimene, 2,6-dimethyl-2-trans-6-octadiene, *β*-springene, 3-hydroxy-*β*-damascone and benzene varied greatly among the three samples, and might serve as markers of differences among the three samples.

In summary, the differences in the substances in the three samples might have contributed to their flavor differences. However, the detailed mechanism of the effect required to be determined by further analysis, such as the analysis of aroma contribution.

### 3.2 Relative odor activity value analysis

In order to explore the aroma contribution of each VOCs to the flavor of the three cigar smoke samples, ROAVs were calculated (**Supplementary Table S2**). Thresholds were selected as the odor thresholds for each compound in air (Gemert, 2011). VOCs with the ROAV larger than 1 contributes more to the overall aroma of the samples and are the key flavor compounds in the samples (Zhu et al., 2020). In addition, VOCs with ROAV between 0.1 and 1 also contributes to the overall aroma of the sample. If the ROAV of a VOC is less than 0.1, it is considered to have a very limited contribution to the overall aroma of the sample.

**Figure 4:**
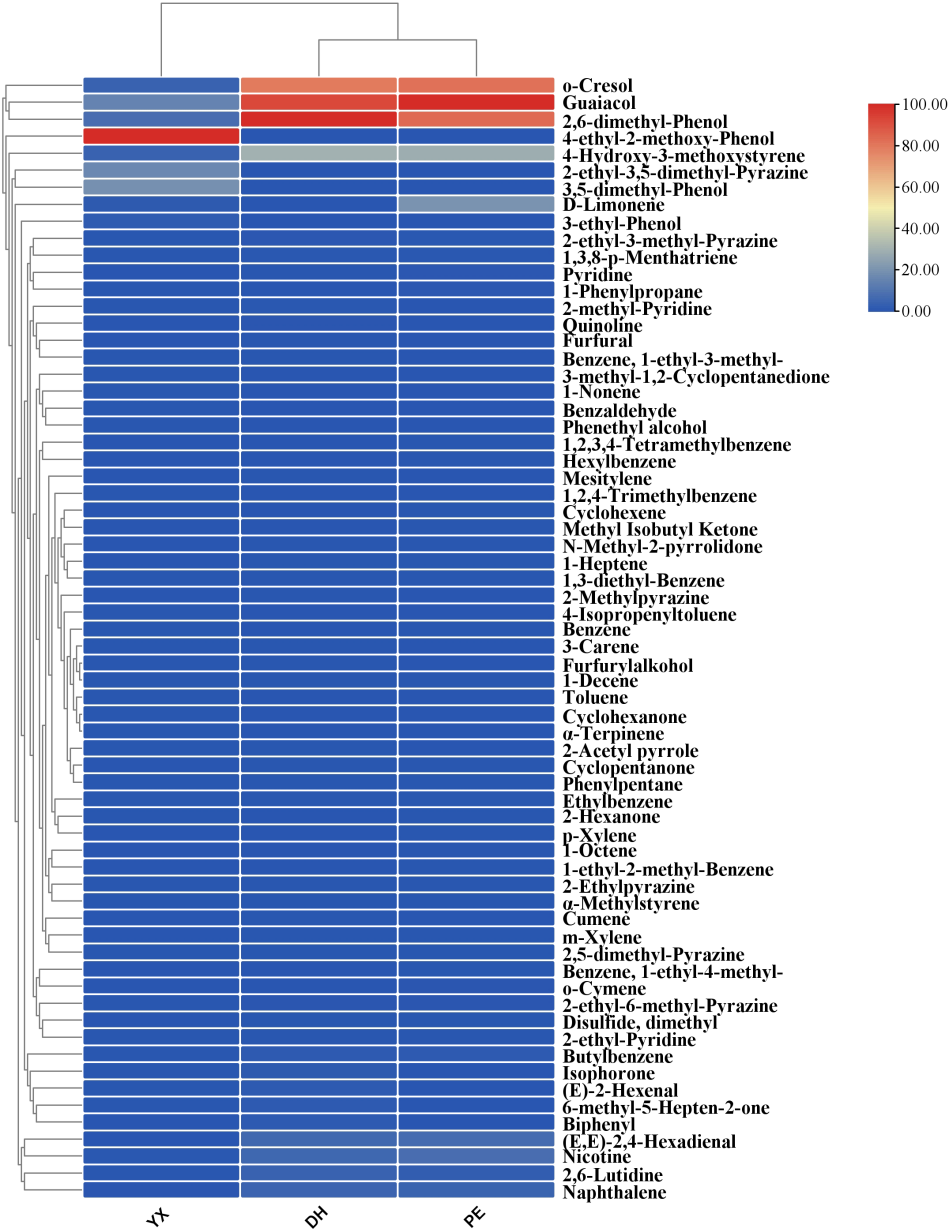
ROAV of each flavor compound in three cigar smoke samples.

In YX, DH and PE, there were 9,13 and 10 VOCs with ROAV > 1, respectively. *o*-Cresol, guaiacol, 2,6-dimethyl-phenol, and 4-hydroxy-3-methoxystyrene had ROAVs greater than 1 in cigar smoke samples from all the three regions and might be the key compounds in the generation of the characteristic flavor of cigar smoke. Additionally, *(E,E)*-2,4-hexadienal, butylbenzene, naphthalene, and nicotine had ROAV > 1 in DH and PE; and D-limonene had ROAV > 1 in YX and PE. These compounds would also like to be a key part of the formation of cigar flavor. 2-Ethyl-3,5-dimethyl-pyrazine, 3-ethyl-phenol, 3,5-dimethyl-phenol and 4-ethyl-2-methoxy-phenol had ROAV > 1 in YX only. *(E)*-2-Hexenal, 6-methyl-5- hepten-2-one, isophorone and biphenyl had ROAV > 1 in DH only. As the consequence of that, these compounds might be the differential flavor compounds that form the characteristic flavor of cigar smoke in different region. To compare the ROAV of the 3 cigar smoke samples more visually, clustered heat map has been drawn (**Figure 4**). The cluster analysis indicated that DH and PE were more similar in terms of aroma contribution, which was consistent with the results of the content analysis. From the differences in color, o-cresol, guaiacol, 2,6-dimethyl-phenol and 4-ethyl-2-methoxy-phenol were particularly important for flavor formation in each sample. In summary, the samples from the three regions had commonalities regarding the contribution of each component to the overall flavor. At the same time, there were also differentiating components that could significantly differentiate the flavor of cigar smoke from other regions.

### 3.3 OPLS-DA modelling and model evaluation of potential differential markers in cigar smoke

**Figure 5:**
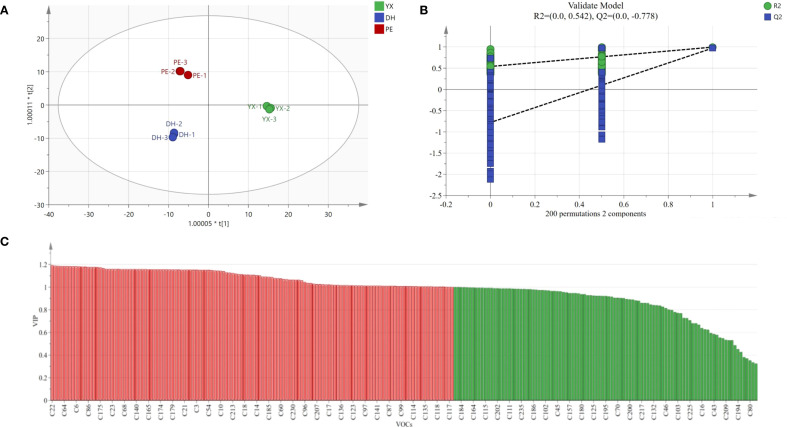
OPLS-DA model of 3 kinds of cigar smoke samples using GC×GC-TOFMS. **(A)** Plot of OPLS-DA scores (R^2^X=0.935, R^2^Y=0.996 and Q^2^ = 0.981). **(B)** Plot of cross-validation of 200 substitution test. **(C)** VIP plots of compound (The red part represents the key differential compounds with VIP > 1).

In order to further screen out potential differential compounds in cigar smoke samples, the data of GC×GC-TOFMS were analyzed by OPLS-DA. As shown in **Figure 5A**, the 3 groups of cigar smoke samples were well clustered on the scatter plot of OPLS-DA scores, with small intra-group differences, and the samples between different groups were completely separated. Both R^2^ and Q^2^ values are higher than 0.5, indicating a strong fit for OPLS-DA model (Yan et al., 2025). As the consequence of this, the model was appropriate for distinguishing cigar smoke from three different production regions, in which R^2^X=0.935, R^2^Y=0.996 and Q^2^ = 0.981. At the same time, the reliability of the model was verified by the replacement test function in SIMCA14.1, and the results of the replacement test after 200 cross-validations have been shown in **Figure 5B**. The Q^2^ regression line intercept was determined to be negative, reinforcing the model’s robustness and validation strength (Yan et al., 2025).

For the OPLS-DA model, variable importance in projection (VIP) is an important parameter to measure the variance components (Zheng et al., 2025). Among the 235 VOCs that were detected, a total of 134 VOCs had a VIP > 1 (**Figure 5C**). The 10 compounds with the largest VIP values (VIP>1.18) were 2-methyl-pyridine (22), 2,4-dimethylfuran (5), 2-carene (88), 13-heptadecyn-1-ol (232), 1-ethyl-2-methyl-benzene (64), 2,3,5,8-tetramethyl-1,5,9-decatriene (203), 1-ethyl-4-methyl-benzene (67), *β*-elemene (199), methyl isobutyl ketone (6) and 3-methylcyclopentanone (29). The above compounds could be used as differential volatile compounds to distinguish cigar smoke from different regions.

### 3.4 Precursors of main flavor compounds in cigar smoke

**Figure 6:**
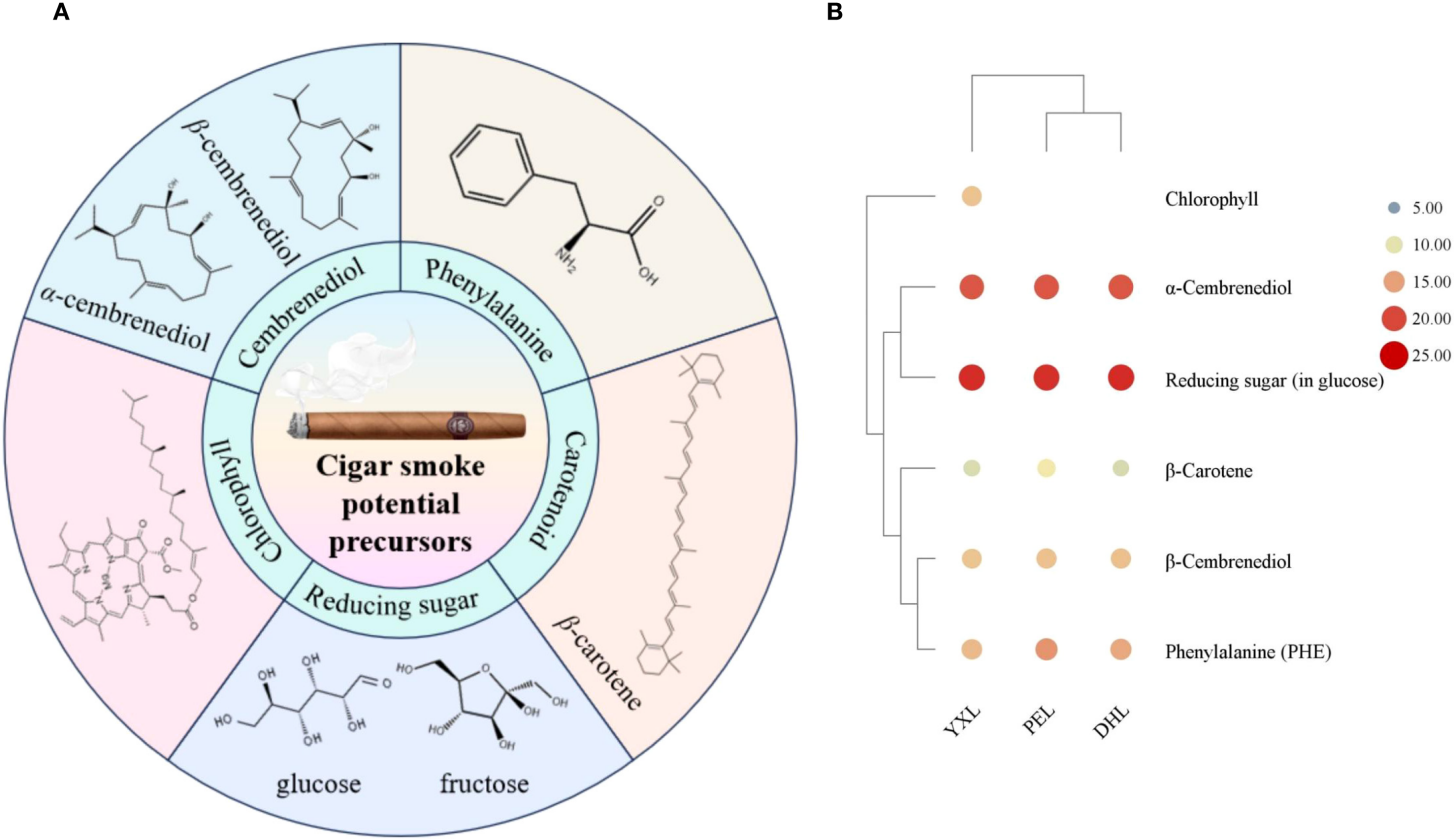
Potential precursors of volatile flavor compounds in cigar smoke. **(A)** Five groups of potential precursors of volatile flavor compounds in cigar smoke. **(B)** The content of potential precursors of volatile flavor compounds in cigar smoke.

In order to further determine the origin of the major flavor compounds in cigar smoke, potential precursors were determined. Based on the formation pathway, the precursors have been divided into 5 main groups: cembrenediol, phenylalanine, chlorophyll, carotenoid, and reducing sugar. The result of the above precursors was shown in **Table 1**. The cigar smoke flavor compounds generated by the degradation of each precursor are summarized in **Figure 6A**.

**Table 1:** Precursors of cigar smoke flavor compounds.

Precursors	Sample			Unit	Standard curve	R2
	PEL	YXL	DHL			
α-Cembrenediol	568084.53 ± 83693.1	573850.07 ± 34667.87	564459 ± 41629.98	*μ*g/kg	y = 1301.777004x-1183.202948	0.9997272
β-Cembrenediol	9538.2 ± 1304.33	8739.47 ± 424.9	10080.8 ± 285.23		y = 24701.977038x-15423.603856	0.99917395
PHE	53293.33 ± 908.3	12605 ± 940.2	24861.67 ± 1052.45		y =52565.2x+4517.56	0.9999569
Chlorophyll	0	8844.67 ± 168.94	0		y =4581.61x-362.671	0.999906
β-Carotene	2224.67 ± 46.7	729.33 ± 17.01	654.67 ± 25.01		y =21076.4x-3925.05	0.9994543
Glucose	11.65 ± 0.1	12.39 ± 0.08	13.74 ± 0.11	g/100g	–	–
Fructose	10.92 ± 0.11	11.62 ± 0.06	12.97 ± 0.09		–	–
Lactose	0.25 ± 0.02	0.22 ± 0.02	0.26 ± 0.02		–	–
Maltose	0.65 ± 0.06	0.74 ± 0.05	0.78 ± 0.11		–	–
Reducing sugar (in glucose)	25.7 ± 0.9	27.97 ± 0.64	31.27 ± 1.4		–	–

“-” indicated that a standard curve was not performed because of the experimental methods.

#### 3.4.1 Cembranoid

Cembranoids are important diterpene compounds in tobacco. Cembrenediol can be categorized by its chemical structure into *α*-cembrenediol and *β*-cembrenediol. For cembrenediol, cigar tobacco was dominated by *α*-cembrenediol, the proportion of which reached more than 98% in DHL, YXL and PEL. Therefore, in terms of content, the flavor compounds in cigar smoke were mainly derived from the degradation of *α*-cembrenediol. This finding was consistent with the findings of previous studies. It has been demonstrated that most cembranoids are derived from the biodegradation of cembrene-4,6-diol (Wahlberg and Enzell, 1987). For cigar smoke, cembrene, solanone, benzaldehyde, styrene may originate from the degradation of *α*-cembrenediol. First, cembrenediol might undergo an elimination reaction to form cembrene. Second, cembrenediol could be degraded by microorganisms to produce solanone. In particular, solanone has a vanilla, tea-like aroma and is an important flavor substance in tobacco (Wang et al., 2014). During tobacco combustion, cembrane degradation products might undergo further pyrolysis to form small molecule compounds such as styrene and benzaldehyde.

#### 3.4.2 PHE

PHE, as an important precursor of tobacco aroma components, can be decomposed to produce benzaldehyde, phenylethanol and other volatile small molecule compounds. These degradation products contributed to the fruity aroma and fresh aroma of the tobacco, which had a direct impact on the formation of the aroma style of the tobacco. In the 3 cigar tobacco samples, the PHE content varied greatly, with PEL having the highest PHE content of 53,293.33 *μ*g/kg. while in YXL, the PHE content was the lowest, at only 12,605.00 *μ*g/kg. the PHE content of PEL, was about 4.2 times higher than that of YXL. As an aromatic free amino acid, the metabolic pathway of phenylalanine can be divided into three main pathways (Yin et al., 2022). First, deamination, in which PHE is converted to trans-cinnamic acid catalyzed by phenylalanine deaminase (PAL), leading to the metabolic generation of secondary substances such as coumarins, lignins, alkaloids and flavonoids. Second, decarboxylation, in which PHE generates intermediates such as phenylethylamine and 2-phenylethylamide by the action of amino acid decarboxylase and peroxidase, which in turn generates benzaldehyde and phenylethanol. Third, PHE undergoes a Meladic reaction with reducing sugars to get heterocyclic compounds such as 2-acetylfuran.

#### 3.4.3 Chlorophyll

As is known to us all, chlorophyll is a class of green pigments contained in higher plants and all other organisms capable of photosynthesis. However, it is also one of the potential precursors for the formation of volatile flavor compounds in tobacco. It was astonishing that the presence of chlorophyll was detected only in YXL among the three cigar tobaccos, with a content of 8,844.67 *μ*g/kg. It’s hypothesized that this phenomenon may be due to the fact that chlorophyll degradation in PEL and DHL was sufficiently high that the detection limit was not reached. In addition, the chlorophyll content is also related to the environment in which the cigars have been grown and the conditions under which they have been processed. Chlorophyll degradation is an important biochemical reaction during tobacco maturation, conditioning and combustion. Among its products, neophytadiene and phytol (herbal, green and floral) play a key role in the aroma quality of tobacco. First, chlorophyll could be hydrolyzed to phytol by chlorophyllolytic metabolizing enzymes. Thereafter, phytol was dehydrated to produce neophytadiene (Changi et al., 2012). In particular, phytone and phytyl acetate detected in the cigar smoke system were also most likely degradation products of chlorophyll. The mechanism of their production still requires further exploration.

#### 3.4.4 Carotenoids

Carotenoids are a group of important natural pigments, including *β*-carotene, lutein, etc. As well as chlorophyll, carotenoids are a class of pigments found in cigars. At the same time, they are also potential precursors for the formation of volatile flavor compounds. Among the multiple carotenoids, *β*-carotene might be the main source of volatile flavor compounds in cigar smoke. The highest amount of *β*-carotene was found in PEL (2224.67 *μ*g/kg), which was approximately more than three times that found in YXL and DHL. In YXL and DHL, the levels of *β*-carotene were equivalent. The generation of carotenoid degradation products mainly involved multi-stage transformation processes in tobacco processing, including tobacco drying, fermentation, maturation and other process. For the three cigar smoke samples in this study, benzaldehyde and 6-methyl-5-hepten-2-one might originate from the degradation of carotenoids (Zhang et al., 2017). In the process of tobacco fermentation, carotenoids are gradually degraded under the effect of lipoxygenase (LOX), peroxidase (POD) and other enzymes, which can generate farnesyl acetone, megastigmatrienone and so on. After that, it is further degraded into small molecule compounds such as *β*-ionone and *β*-damascone (Zhang et al., 2017). However, these compounds were not detected in this study, which may be related to the assay method and the samples themselves. During the conversion of cigar combustion to smoke, the degradation products might be further pyrolyzed to form benzaldehyde and 6-methyl-5-hepten-2-one as detected herein.

#### 3.4.5 Reducing sugar

The Maillard reaction, a reaction between carbonyl compounds (reducing sugars) and amino compounds (amino acids and proteins), is a non-enzymatic browning reaction widespread in the food industry. The reducing sugars in cigar tobacco can undergo a Maillard reaction with amino acids, such as the aforementioned PHE. There was a minimal difference in the level of reducing sugar content among the three samples, with the highest reducing sugar content in DHL (31.27 g/100g) and the lowest in PEL (25.7 g/100g). The reducing sugars in cigar tobacco were dominated by glucose and fructose, and they accounted for more than 85% of the total reducing sugars. The possible products of the Maillard reaction in the 3 regional cigars include furfural, furfurylalkohol, 2-acetyl pyrrole and 2,3’-dipyridyl. These products have given the cigar a roasted, burnt and smoky aroma.

Overall, reducing sugars were the most abundant of the five groups of precursors, followed by *α*-cembrenediol, PHE, *β*-cembrenediol, chlorophyll, and *β*-carotene (**Figure 6B**). Reducing sugars, as one of the primary reactants of the Maillard reaction, might promote the generation of Maillard reactions and the formation of Maillard products. Specifically, it might promote the formation of heterocyclic compounds which are important for smoke flavor. And this result was confirmed by the finding that cigar smoke was rich in heterocyclic compounds.

In a nutshell, the flavor compounds in cigars mainly originate from five major groups of potential precursor components: cembrenediol, phenylalanine, chlorophyll, carotenoid and reducing sugar. The exact degradation routes still need to be explored and verified by methods such as modeling experiments and isotope tracer techniques.

## 4 Conclusions

In this study, we explored the mechanisms of the flavor formation of cigar smoke in three regions (YX, DH and PE) by means of GC×GC-TOFMS, HPLC, Multivariate Statistical Analysis, and ROAV analysis. A total of 235 volatile compounds were identified in the three samples by GC×GC-TOFMS, including 72 alkenes, 47 aromatic hydrocarbons, 42 heterocyclics, 28 ketones, 8 alcohols, 4 aldehydes, 3 esters, 13 phenols, 5 acetonitrile and 13 others. The results of the ROAV analysis showed that 9 (YX), 13 (DH) and 10 (PE) VOCs contributed significantly to the overall aroma of cigar smoke in the three regions, respectively. o-Cresol, guaiacol, 2,6-dimethylphenol, and 4-hydroxy-3-methoxystyrene had ROAVs greater than 1 in all three samples, suggesting these compounds might be key flavor compounds which could represent the flavor characteristics of cigar smoke. Next, differential volatile compounds were determined for the individual cigar smoke samples by OPLS-DA. We hypothesized that the differences in cigar smoke VOCs, key flavor compounds, and overall aroma across the three regions might be explained by the growing environment, such as sunlight duration, rainfall, and temperature. Finally, the potential precursors of flavor compounds in cigar smoke were analyzed. The precursors were categorized into five main groups, with reducing sugars being the most abundant in cigar leaves.

This paper provided a preliminary analysis of the mechanisms of cigar flavor formation from the perspective of volatile flavor compounds and their precursors, as well as the flavor variations of cigar smoke in three regions in Yunnan (Yuxi, Dehong, and Pu’er). The above studies have provided theoretical guidance for process optimization of cigar flavors and flavor stabilization in industry. However, this study also has certain limitations in its research methods and data collection. For example, the signal-to-noise ratio may affect the accuracy of the concentrations of various volatile compounds. In the future, we can improve the determination of VOCs in cigar smoke by using more detection methods and enhance the representativeness of the data by increasing the sample number. Besides that, it will be possible to conduct more accurate quantitative analyses of key flavor compounds in cigars, such as Stable Isotope Dilution Analysis (SIDA). In addition, modeling experiments, isotope tracing and other techniques can be used to determine the pathways of flavor compounds in order to regulate the flavor of cigar smoke from the source.

## Data Availability

The original contributions presented in the study are included in the article/**Supplementary Material**. Further inquiries can be directed to the corresponding authors.
