# Supplementary material for: Unraveling flavor formation mechanism of cigar smoke through volatile compounds in cigar smoke and potential precursors in cigar tobacco
Source: Front Plant Sci. 2025 Sep 25;16:1672119. doi: 10.3389/fpls.2025.1672119 (PMC12509428; doi:10.3389/fpls.2025.1672119)
Supplement: Supplementary file 1 [file DataSheet1.docx]

Table S1 Volatile compounds and their contents in dfifferent cigar smoke.

| No. | 1D RT (min) | 2D RT (min) | Compound | CAS | Content (μg/L) | | | RI | NIST RI | Identification |
| --- | --- | --- | --- | --- | --- | --- | --- | --- | --- | --- |
|  |  |  |  |  | YX | DH | PE |  |  |  |
| 1 | 6.3596 | 2.816 | Benzene | 71-43-2 | 54.29±3.87 | - | 7.88±0.88 | - | 654-S | MS |
| 2 | 6.5925 | 0.621 | 3-Penten-2-one | 625-33-2 | - | 4.65±2.17 | - | - | 733-S | MS |
| 3 | 6.8255 | 0.518 | Cyclohexene | 110-83-8 | - | - | 3.82±0.73 | - | 679-S | MS |
| 4 | 7.059 | 0.432 | 1-Heptene | 592-76-7 | - | 6.28±1.03 | 8.03±2.35 | - | 685-S | MS |
| 5 | 7.7595 | 0.663 | 2,4-Dimethylfuran | 3710-43-8 | 2.38±0.58 | 3.13±0.11 | - | 711 | 729-S | MS, RI |
| 6 | 8.5757 | 0.839 | Methyl Isobutyl Ketone | 108-10-1 | - | - | 8.93±1.18 | 731 | 735-S | MS, RI |
| 7 | 8.8092 | 1.188 | Pyridine | 110-86-1 | - | 15.12±0.92 | 39.89±5.89 | 736 | 746-S | MS, RI |
| 8 | 8.9258 | 0.992 | Disulfide, dimethyl | 624-92-0 | - | 5.47±0.53 | 5.24±0.79 | 739 | 746-S | MS, RI |
| 9 | 9.1594 | 0.639 | (1-methylethylidene)-Cyclobutane | 1528-22-9 | 10.94±1.29 | - | - | 745 | - | MS, RI |
| 10 | 9.3923 | 0.559 | cis-1,2,trans-1,3-1,2,3-Trimethylcyclopentane in cyclohexane | 15890-40-1 | - | 2.26±0.46 | - | 750 | 775-S | MS, RI |
| 11 | 9.6259 | 0.74 | 2,3-dimethyl-1,3-Pentadiene | 1113-56-0 | - | 2.82±0.12 | - | 756 | - | MS, RI |
| 12 | 9.8596 | 1.014 | Toluene | 108-88-3 | 20.75±1.43 | - | - | 761 | 763-S | MS, RI |
| 13 | 10.0924 | 0.745 | 1-methyl-Cyclohexene | 591-49-1 | - | 3.04±1.28 | - | 767 | 766-S | MS, RI |
| 14 | 10.4423 | 0.64 | 6-methyl-2-Heptene | 73548-72-8 | - | 1.64±0.57 | 4.88±0.72 | 775 | 787-S | MS, RI |
| 15 | 10.5593 | 0.901 | 1-methyl-1,4-Cyclohexadiene | 4313-57-9 | 3.56±1.36 | 2.4±0.1 | 2.7±0.23 | 778 | 790-S | MS, RI |
| 16 | 10.6763 | 1.043 | 3-Hexanone | 589-38-8 | 5.07±1.46 | 6.67±0.2 | 6.08±1.18 | 781 | 784-S | MS, RI |
| 17 | 10.7926 | 0.644 | 2-methyl-1-Heptene | 15870-10-7 | - | 3.09±0.12 | 3.49±0.41 | 783 | 776-S | MS, RI |
| 18 | 10.9096 | 1.118 | 2-Hexanone | 591-78-6 | 3.82±1.02 | - | 4.55±1.22 | 786 | 790-S | MS, RI |
| 19 | 11.0256 | 0.657 | 1-Octene | 111-66-0 | - | - | 12.46±1.76 | 789 | 789-S | MS, RI |
| 20 | 11.0258 | 1.557 | Cyclopentanone | 120-92-3 | - | 19.02±0.96 | 19.43±6.38 | 789 | 791-S | MS, RI |
| 21 | 12.1925 | 1.499 | 1-Ethylpyrrole | 617-92-5 | - | 4.62±0.62 | - | 809 | 821-S | MS, RI |
| 22 | 12.4261 | 1.636 | 2-methyl-Pyridine | 109-06-8 | 13.46±1.92 | 18.07±1.89 | - | 812 | 818-S | MS, RI |
| 23 | 13.1259 | 1.839 | 2-Methylpyrazine | 109-08-0 | - | 11.76±0.43 | - | 821 | 831-S | MS, RI |
| 24 | 13.5924 | 2.215 | Furfural | 98-01-1 | - | 0.79±0.45 | - | 827 | 833-S | MS, RI |
| 25 | 14.2929 | 1.703 | (E)-2-Hexenal | 6728-26-3 | 31.92±5.32 | 28.19±0.64 | - | 836 | 854-S | MS, RI |
| 26 | 14.4091 | 1.973 | 3-Methylpyrrole | 616-43-3 | - | 10.22±1.18 | 5.88±0.11 | 838 | 858-S | MS, RI |
| 27 | 14.8756 | 0.79 | 2,6-dimethyl-3-Heptene | 2738-18-3 | - | 6.6±0.12 | - | 843 | - | MS, RI |
| 28 | 14.876 | 0.781 | 2,6-dimethyl-1-Heptene | 3074-78-0 | 6.75±1.46 | - | - | 843 | 866-S | MS, RI |
| 29 | 14.8754 | 1.847 | 3-Methylcyclopentanone | 6672-30-6 | - | - | 7.85±1.13 | 844 | 848-S | MS, RI |
| 30 | 15.1096 | 2.098 | Furfurylalkohol | 98-00-0 | 6.9±1.31 | 3.41±0.63 | - | 847 | 859-S | MS, RI |
| 31 | 15.4595 | 1.166 | Lurasidone | 4430-91-5 | 1.84±0.29 | - | - | 851 | 856-S | MS, RI |
| 32 | 15.6924 | 0.938 | 1,2,4,4-Tetramethylcyclopentene | 65378-76-9 | - | 4.67±0.22 | 5.24±0.58 | 854 | 857-S | MS, RI |
| 33 | 15.6928 | 2.044 | 3-Picoline | 108-99-6 | 51.08±7.24 | 31.11±20.71 | 62.73±6.13 | 854 | 863-S | MS, RI |
| 34 | 15.8094 | 1.428 | Ethylbenzene | 100-41-4 | 20.58±1.84 | 20.39±0.97 | 26.06±1.66 | 855 | 855-S | MS, RI |
| 35 | 16.2759 | 2.357 | (E,E)-2,4-Hexadienal | 142-83-6 | - | 8.85±0.77 | 10.24±1.96 | 862 | 911-S | MS, RI |
| 36 | 16.6258 | 1.401 | m-Xylene | 108-38-3 | - | 41.04±2.44 | 38.18±20.51 | 866 | 866-S | MS, RI |
| 37 | 17.0923 | 1.932 | Phenylethyne | 536-74-3 | - | 1.49±0.12 | - | 872 | 834-S | MS, RI |
| 38 | 17.5596 | 2.107 | 2,6-Lutidine | 108-48-5 | 9.12±2.73 | 9.11±0.3 | 5.95±2.07 | 878 | 878-S | MS, RI |
| 39 | 18.4929 | 0.879 | 1-Nonene | 124-11-8 | 3.75±0.71 | - | - | 890 | 889-S | MS, RI |
| 40 | 18.4923 | 1.636 | p-Xylene | 106-42-3 | - | - | 12.28±1.93 | 890 | 865-S | MS, RI |
| 41 | 18.4926 | 1.843 | 1,3,5,7-Cyclooctatetraene | 629-20-9 | - | 26.4±5.07 | - | 890 | 850-S | MS, RI |
| 42 | 18.7262 | 2.456 | Cyclohexanone | 108-94-1 | 2.76±0.56 | - | - | 893 | 894-S | MS, RI |
| 43 | 19.426 | 2.302 | 2-ethyl-Pyridine | 100-71-0 | 9.83±1.86 | 8.23±0.4 | 7.39±2.31 | 901 | 906-S | MS, RI |
| 44 | 19.543 | 2.659 | 2-Cyclopenten-1-one, 2-methyl- | 1120-73-6 | 36.71±5.69 | 28.37±17.67 | 35.67±1.24 | 902 | 913-S | MS, RI |
| 45 | 19.6596 | 0.989 | 3,3,6-trimethyl-1,5-Heptadiene | 35387-63-4 | 8.21±1.82 | 2.01±0.08 | 3.95±0.89 | 903 | 904-S | MS, RI |
| 46 | 20.0095 | 2.804 | Ethanone, 1-(2-furanyl)- | 1192-62-7 | 7.99±1.31 | 4.98±0.3 | 5.16±1.57 | 907 | 911-S | MS, RI |
| 47 | 20.2427 | 0.883 | 3,7-dimethyl-1-Octene | 4984-01-4 | 2.45±0.36 | 1.99±0.43 | 2.05±0.65 | 908 | - | MS, RI |
| 48 | 20.3594 | 2.39 | 2,5-dimethyl-Pyrazine | 123-32-0 | 14.4±5.25 | 9.78±0.95 | 9.56±1.53 | 910 | 917-S | MS, RI |
| 49 | 20.7096 | 2.427 | 2-Ethylpyrazine | 13925-00-3 | 9.77±2.03 | 6.46±0.58 | 7.4±1.44 | 913 | 921-S | MS, RI |
| 50 | 20.8262 | 1.077 | 1,5,5-trimethyl-3-methylene-Cyclohexene | 16609-28-2 | 9.41±1.59 | - | - | 914 | - | MS, RI |
| 51 | 21.6423 | 1.722 | Cumene | 98-82-8 | - | 1.43±0.08 | - | 921 | 921-S | MS, RI |
| 52 | 21.8759 | 2.615 | 2,4-dimethyl-Pyridine | 108-47-4 | - | 9.66±2.9 | - | 923 | 930-S | MS, RI |
| 53 | 22.1095 | 2.505 | 2,5-dimethyl-Pyridine | 589-93-5 | 25.92±9.98 | 13.08±0.5 | - | 925 | 922-S | MS, RI |
| 54 | 22.9263 | 1.566 | 2-methyl-3-Heptanone | 13019-20-0 | 36.62±5.6 | - | 39.94±4.68 | 932 | - | MS, RI |
| 55 | 23.2762 | 2.298 | 2-ethyl-Cyclopentanone | 4971-18-0 | 7.4±1.14 | 6.16±0.17 | 6.43±0.51 | 936 | - | MS, RI |
| 56 | 23.8593 | 1.129 | β-Citronellene | 10281-56-8 | 6.42±1.09 | - | - | 941 | 943-S | MS, RI |
| 57 | 23.9763 | 1.988 | Allylbenzene | 300-57-2 | 3.97±0.56 | - | - | 942 | 934-S | MS, RI |
| 58 | 24.4428 | 1.19 | (1α,3α,6α)-3,7,7-trimethyl-Bicyclo[4.1.0]heptane | 18968-23-5 | 2.89±0.4 | - | - | 946 | 978-S | MS, RI |
| 59 | 24.4424 | 1.676 | 3-methyl-Pentanoic acid | 105-43-1 | - | 17.57±2.24 | - | 946 | 947-S | MS, RI |
| 60 | 24.7927 | 1.824 | 1-Phenylpropane | 103-65-1 | 11.7±1.85 | - | 8.63±1.79 | 949 | 953-S | MS, RI |
| 61 | 25.0263 | 2.739 | 3-ethyl-Pyridine | 536-78-7 | 23.94±6.36 | 17.08±3.29 | 17.08±6.25 | 951 | 959-S | MS, RI |
| 62 | 25.6095 | 1.873 | 1-ethyl-3-methyl-Benzene | 620-14-4 | 23.57±3.7 | 11.69±0.24 | - | 956 | 957-S | MS, RI |
| 63 | 25.6091 | 2.443 | 2-ethyl-6-methyl-Pyridine | 1122-69-6 | - | 10.76±1.1 | - | 956 | 971-S | MS, RI |
| 64 | 25.6089 | 1.912 | 1-ethyl-2-methyl-Benzene | 611-14-3 | - | - | 13.25±1.46 | 956 | 970-S | MS, RI |
| 65 | 25.6091 | 3.575 | 3-methyl-2-Cyclopenten-1-one | 2758-18-1 | - | 24.19±2.14 | - | 957 | 972-S | MS, RI |
| 66 | 25.7261 | 3.117 | Benzaldehyde | 100-52-7 | 20.3±6.09 | - | - | 958 | 962-S | MS, RI |
| 67 | 25.9587 | 1.904 | 1-ethyl-4-methyl-Benzene | 622-96-8 | - | - | 7.63±0.9 | 960 | 954-S | MS, RI |
| 68 | 26.6591 | 1.829 | Mesitylene | 108-67-8 | - | 3.86±0.26 | - | 966 | 972-S | MS, RI |
| 69 | 27.7095 | 1.425 | 2-methyl-6-methylene-1,7-Octadiene | 1686-30-2 | 12.86±2.79 | - | 3.17±0.49 | 975 | 984-S | MS, RI |
| 70 | 28.0594 | 2.271 | α-Methylstyrene | 98-83-9 | 6.35±1.26 | 3.55±0.07 | 4.14±0.43 | 978 | 976-S | MS, RI |
| 71 | 28.176 | 1.553 | 1,5,5,6-tetramethyl-1,3-Cyclohexadiene | 514-94-3 | 9.99±3.95 | - | - | 979 | - | MS, RI |
| 72 | 28.2926 | 3.497 | Benzonitrile | 100-47-0 | 14.49±3.32 | 15.55±0.9 | 18.95±0.85 | 981 | 985-S | MS, RI |
| 73 | 28.6429 | 1.283 | 2,6-dimethyl-2-trans-6-octadiene | 2492-22-0 | 30.8±6.44 | - | 9.11±1.47 | 983 | - | MS, RI |
| 74 | 28.6429 | 2.246 | 6-methyl-5-Hepten-2-one | 110-93-0 | 11.1±1.97 | 5.94±0.24 | - | 984 | 986-S | MS, RI |
| 75 | 28.9928 | 3.22 | 3,4-dimethylcyclopent-2-en-1-one | 30434-64-1 | 22.04±4.96 | 17.01±2.08 | 14.52±2.71 | 987 | 986-S | MS, RI |
| 76 | 29.3427 | 1.599 | 2,6-dimethyl-3,5-Heptadien-2-ol | 77411-76-8 | 13.62±0.46 | - | - | 990 | - | MS, RI |
| 77 | 29.4596 | 1.08 | 1-Decene | 872-05-9 | 7.64±1 | 7.55±0.32 | 8.39±1.3 | 991 | 989-S | MS, RI |
| 78 | 29.6929 | 2.248 | 2-Methylstyrene | 611-15-4 | 25.26±3.22 | 13.83±0.42 | - | 993 | 975-S | MS, RI |
| 79 | 29.9261 | 2.816 | 2-ethyl-6-methyl-Pyrazine | 13925-03-6 | 11.17±2.36 | 9.78±0.31 | 9.82±2.13 | 995 | 1003-S | MS, RI |
| 80 | 30.3927 | 2.858 | 2-ethyl-3-methyl-Pyrazine | 15707-23-0 | 15.89±5.67 | 14.43±0.48 | 13.68±0.98 | 999 | 1004-S | MS, RI |
| 81 | 30.7425 | 2.792 | 2,4,6-Collidine | 108-75-8 | - | 4.6±1.64 | - | 1002 | 991-S | MS, RI |
| 82 | 30.8591 | 2.995 | 2-ethyl-4-methyl-1H-Pyrrole | 69687-77-0 | - | 5.77±0.65 | - | 1003 | 938-S | MS, RI |
| 83 | 30.9762 | 4.411 | 3-Pyridinecarbonitrile | 100-54-9 | 4.37±1.13 | 3.34±0.34 | - | 1004 | 1012-S | MS, RI |
| 84 | 31.2094 | 1.571 | 3-Carene | 13466-78-9 | 12.25±1.48 | 3.53±0.94 | - | 1005 | 1011-S | MS, RI |
| 85 | 32.0262 | 1.595 | 2,5,6-Trimethyl-1,3,6-heptatriene | 42123-66-0 | 53.07±6.63 | 9.81±1.01 | - | 1011 | - | MS, RI |
| 86 | 32.2588 | 1.107 | 4-methyl-1-Decene | 13151-29-6 | - | - | 6.61±1.19 | 1013 | - | MS, RI |
| 87 | 32.4927 | 1.624 | α-Terpinene | 99-86-5 | 8.64±1.3 | - | - | 1015 | 1017-S | MS, RI |
| 88 | 32.4921 | 1.683 | 2-Carene | 554-61-0 | - | - | 0.98±0.08 | 1015 | 1001-S | MS, RI |
| 89 | 32.8423 | 2.091 | o-Cymene | 527-84-4 | - | - | 0.97±0.26 | 1018 | 1022-S | MS, RI |
| 90 | 32.8429 | 2.264 | 1,2,4-Trimethylbenzene | 95-63-6 | 25.05±3.7 | - | - | 1018 | 990-S | MS, RI |
| 91 | 32.9596 | 3.555 | 3-methyl-1,2-Cyclopentanedione | 765-70-8 | 28.48±5.78 | - | - | 1019 | 1043-S | MS, RI |
| 92 | 33.3091 | 1.442 | p-Menth-1-ene | 1195-31-9 | - | 15.16±0.65 | 23.61±1.9 | 1021 | - | MS, RI |
| 93 | 33.4261 | 1.836 | 1,3,5-tris(methylene)-Cycloheptane | 68284-24-2 | 34.09±15.18 | - | - | 1022 | - | MS, RI |
| 94 | 34.0096 | 1.541 | D-Limonene | 5989-27-5 | 126.18±12.07 | - | 93.7±5.68 | 1026 | 1035 | MS, RI |
| 95 | 34.3591 | 3.621 | 2,3-dimethyl-2-Cyclopenten-1-one | 1121-05-7 | 30.36±4.49 | 33.46±1 | 34.22±3.69 | 1029 | 1040-S | MS, RI |
| 96 | 34.3591 | 5.218 | N-Methyl-2-pyrrolidone | 872-50-4 | - | 49.97±3.76 | 70.05±4.39 | 1029 | 1044-S | MS, RI |
| 97 | 34.4761 | 2.403 | 1,2,3-Trimethylbenzene | 526-73-8 | 5.45±0.75 | - | - | 1030 | 1013-S | MS, RI |
| 98 | 35.0592 | 1.021 | 2,7,10-trimethyl-Dodecane | 74645-98-0 | - | 0.78±0.01 | - | 1034 | - | MS, RI |
| 99 | 35.4095 | 1.625 | 3,8-p-Menthadiene | 586-67-4 | 13.29±2.17 | - | - | 1037 | 1075-S | MS, RI |
| 100 | 35.6428 | 3.011 | 3-phenyl-1-Propyne | 10147-11-2 | 17.22±2.3 | 15.28±1.37 | 19.53±3.07 | 1039 | - | MS, RI |
| 101 | 35.7594 | 2.32 | 4-Phenyl-1-butene | 768-56-9 | 2.46±0.56 | 2.29±0.09 | - | 1040 | - | MS, RI |
| 102 | 36.3429 | 2.165 | 1,3-diethyl-Benzene | 141-93-5 | 6.74±1.13 | 1.8±0.1 | 2±0.91 | 1044 | 1032-S | MS, RI |
| 103 | 36.8094 | 2.072 | 3-Propyltoluene | 1074-43-7 | 15.06±12.71 | 3.3±0.15 | - | 1047 | 1037-S | MS, RI |
| 104 | 36.926 | 3.168 | o-Cresol | 95-48-7 | 24.33±2.31 | 24.66±2.95 | 25.82±3.5 | 1049 | 1054-S | MS, RI |
| 105 | 37.5095 | 2.082 | Butylbenzene | 104-51-8 | 22.94±4.03 | 11.93±0.24 | 13.16±2.48 | 1053 | 1054-S | MS, RI |
| 106 | 37.859 | 1.068 | 5-methyldecane | 13151-35-4 | - | 3.78±0.18 | 4.36±1.04 | 1055 | 1057-S | MS, RI |
| 107 | 38.0927 | 4.305 | 2-Acetyl pyrrole | 1072-83-9 | 3.62±1.06 | 1.64±0.19 | - | 1057 | 1064-S | MS, RI |
| 108 | 38.5595 | 2.229 | 2-Propyltoluene | 1074-17-5 | 5.31±1.29 | - | - | 1061 | 1047-S | MS, RI |
| 109 | 38.6758 | 2.824 | 2-ethyl-4,6-dimethyl-Pyridine | 1124-35-2 | - | 1.76±0.15 | - | 1062 | 1080-S | MS, RI |
| 110 | 39.4929 | 3.953 | 3-ethyl-2-Cyclopenten-1-one | 5682-69-9 | 15.83±3.29 | 7.29±5.57 | - | 1068 | 1052-S | MS, RI |
| 111 | 40.7762 | 2.861 | Tetracyclo[5.3.0.0<2,6>.0<3,10>]deca-4,8-diene | 34324-40-8 | 4.76±1.34 | - | - | 1077 | - | MS, RI |
| 112 | 40.7758 | 2.87 | 2-Methylindene | 2177-47-1 | - | 5.27±0.44 | - | 1077 | 1055-S | MS, RI |
| 113 | 40.8928 | 2.464 | m,α-dimethylstyrene | 1124-20-5 | 9.1±2.04 | - | - | 1078 | 1082-S | MS, RI |
| 114 | 41.0095 | 1.791 | 6-isopropylidene-1-methyl-Bicyclo[3.1.0]hexane | 24524-57-0 | 2.15±0.37 | - | - | 1079 | - | MS, RI |
| 115 | 41.2427 | 3.694 | Guaiacol | 90-05-1 | 21.34±2.68 | 7.18±0.55 | 7.88±1.33 | 1081 | 1090-S | MS, RI |
| 116 | 41.3594 | 3.414 | 2,3,4-trimethylcyclopent-2-en-1-one | 28790-86-5 | 21.35±6.01 | - | - | 1082 | 1071-S | MS, RI |
| 117 | 41.3594 | 3.047 | 2-ethyl-3,5-dimethyl-Pyrazine | 13925-07-0 | 2.38±0.51 | - | - | 1082 | 1084-S | MS, RI |
| 118 | 41.476 | 1.969 | Terpinolene | 586-62-9 | 11.6±2.26 | - | - | 1083 | 1088-S | MS, RI |
| 119 | 41.7096 | 2.433 | 2-Ethyl-p-xylene | 1758-88-9 | 9.65±3.17 | - | - | 1084 | 1074-S | MS, RI |
| 120 | 41.709 | 2.485 | 5-Ethyl-m-xylene | 934-74-7 | - | - | 2.56±1.14 | 1084 | 1058-S | MS, RI |
| 121 | 41.8262 | 3.665 | o-Tolunitrile | 529-19-1 | 9.7±5.17 | 8.5±0.48 | 7.49±2.19 | 1085 | 1108-S | MS, RI |
| 122 | 42.1761 | 2.419 | 4-Isopropenyltoluene | 1195-32-0 | 26.04±3.92 | - | - | 1088 | 1090-S | MS, RI |
| 123 | 42.643 | 1.189 | 1-Undecene | 821-95-4 | 17.87±1.04 | 12.28±0.36 | 15.44±1.16 | 1091 | 1091-S | MS, RI |
| 124 | 43.4594 | 2.52 | 4-Ethenyl-1,2-dimethylbenzene | 27831-13-6 | 6.5±3.37 | 3.6±0.26 | - | 1098 | - | MS, RI |
| 125 | 44.1595 | 3.286 | 2,6-dimethyl-Phenol | 576-26-1 | 22.7±3.97 | 15.48±0.75 | 13.19±1.75 | 1103 | 1108-S | MS, RI |
| 126 | 44.7427 | 2.1 | 1-methyl-4-(1-methylpropyl)-Benzene | 1595-16-0 | 6.67±1.05 | - | - | 1107 | 1090-S | MS, RI |
| 127 | 44.8596 | 3.885 | Phenethyl alcohol | 60-12-8 | 14.59±2.63 | - | - | 1108 | 1116-S | MS, RI |
| 128 | 45.2095 | 2.236 | 1,3,8-p-Menthatriene | 18368-95-1 | 18.98±2.17 | 4.8±0.04 | 5.56±1 | 1110 | 1119-S | MS, RI |
| 129 | 45.2095 | 3.623 | Ethylcyclopentenolone | 21835-01-8 | 14.42±7.81 | 3.4±0.24 | - | 1110 | 1106-S | MS, RI |
| 130 | 45.5594 | 1.779 | (E)-4,8-Dimethylnona-1,3,7-triene | 19945-61-0 | 15.01±4.03 | - | - | 1113 | 1116-S | MS, RI |
| 131 | 45.559 | 1.302 | 1-pentyl-2-propyl-Cyclopropane | 41977-33-7 | - | 1.52±0.07 | - | 1113 | - | MS, RI |
| 132 | 46.1429 | 1.766 | 3-(1-methylethenyl)-Cyclooctene | 61233-78-1 | 2.56±1.82 | - | - | 1117 | - | MS, RI |
| 133 | 46.1429 | 3.19 | Isophorone | 78-59-1 | 3.18±0.81 | 1.93±0.18 | - | 1117 | 1124-S | MS, RI |
| 134 | 46.7257 | 2.601 | 2-Methyl-1-phenylpropene | 768-49-0 | - | 9.82±0.95 | - | 1121 | - | MS, RI |
| 135 | 47.0763 | 1.787 | 2-ethenyl-1,1-dimethyl-3-methylene-Cyclohexane | 95452-08-7 | 6.08±1.04 | - | - | 1124 | - | MS, RI |
| 136 | 47.1929 | 2.338 | 1-methyl-3-(1-methyl-2-propenyl)-Benzene | 52161-57-6 | 3.43±0.29 | - | - | 1125 | - | MS, RI |
| 137 | 47.4262 | 1.88 | Neo-alloocimene | 7216-56-0 | 25.78±7.45 | - | 7.04±1.29 | 1126 | 1131-S | MS, RI |
| 138 | 47.5428 | 2.07 | (3E,5E)-2,6-Dimethyl-1,3,5,7-octatetrene | 460-01-5 | 13.24±3.67 | - | - | 1127 | 1131-S | MS, RI |
| 139 | 48.3592 | 5.026 | Benzeneacetonitrile | 140-29-4 | - | 17.32±0.15 | 14.99±1.83 | 1134 | 1144-S | MS, RI |
| 140 | 48.5924 | 2.426 | (3-methyl-3-butenyl)-Benzene | 6683-51-8 | - | 1.08±0.01 | - | 1135 | - | MS, RI |
| 141 | 48.7091 | 3.23 | 2-Ethylphenol | 90-00-6 | - | 15.87±0.48 | 17.63±3.74 | 1136 | 1139-S | MS, RI |
| 142 | 49.0594 | 2.314 | 1-ethyl-4-(1-methylethyl)-Benzene | 4218-48-8 | 12.06±2.23 | - | - | 1138 | 1096-S | MS, RI |
| 143 | 49.6424 | 3.11 | 1,2-Dihydronaphthalene | 447-53-0 | - | 23.28±2.4 | - | 1143 | 1133-S | MS, RI |
| 144 | 49.6424 | 2.898 | 1-Allyl-2-methylbenzene | 1587-04-8 | - | 5.41±1.08 | - | 1143 | - | MS, RI |
| 145 | 49.8757 | 2.761 | 2-Isobutyl-4-methylpyridine | 85665-88-9 | - | 2.95±1.05 | - | 1144 | 1154-S | MS, RI |
| 146 | 49.9921 | 2.646 | 1,2,3,4-Tetramethylbenzene | 488-23-3 | - | - | 5±0.54 | 1145 | 1145-S | MS, RI |
| 147 | 51.159 | 1.141 | 6-methylundecane | 17302-33-9 | - | 0.8±0.01 | - | 1153 | 1152-S | MS, RI |
| 148 | 51.1594 | 2.205 | Phenylpentane | 538-68-1 | 6.31±2.14 | 4.66±0.27 | 5.11±1.11 | 1154 | 1157-S | MS, RI |
| 149 | 51.276 | 0.182 | 6-Ethyl-5,6-dihydro-2H-pyran-2-one | 19895-35-3 | 5.27±0.85 | - | - | 1154 | 1160-S | MS, RI |
| 150 | 52.2088 | 2.358 | 1-methyl-4-butylBenzene | 1595-05-7 | - | - | 4.44±1.91 | 1161 | 1139-S | MS, RI |
| 151 | 52.6759 | 1.116 | 5-propyl-Decane | 17312-62-8 | - | 1.57±0.03 | - | 1164 | - | MS, RI |
| 152 | 52.7925 | 3.27 | 4-Ethylphenol | 123-07-9 | - | 26.92±2.05 | - | 1166 | 1169-S | MS, RI |
| 153 | 53.2594 | 3.249 | 3-ethyl-Phenol | 620-17-7 | 25.99±7.82 | - | - | 1169 | 1169-S | MS, RI |
| 154 | 53.4923 | 1.159 | 3-Methylundecane | 1002-43-3 | - | 0.96±0.05 | - | 1170 | 1170-S | MS, RI |
| 155 | 53.9591 | 2.918 | 2,3-dimethyl-1H-Indene | 4773-82-4 | - | 6.33±0.77 | - | 1174 | - | MS, RI |
| 156 | 54.0758 | 4.188 | 1-Furfurylpyrrole | 1438-94-4 | - | 5.23±0.29 | - | 1175 | 1187-S | MS, RI |
| 157 | 54.1928 | 2.514 | 2,4,6-Trimethylstyrene | 769-25-5 | 8.05±3.41 | - | - | 1176 | - | MS, RI |
| 158 | 54.5427 | 3.766 | Naphthalene | 91-20-3 | 30.9±8.91 | 25.88±0.35 | 25.11±0.57 | 1178 | 1182-S | MS, RI |
| 159 | 55.1258 | 2.626 | 2,2-dimethyl-1,3-dihydroindene | 20836-11-7 | - | 6.1±0.92 | - | 1182 | - | MS, RI |
| 160 | 55.3595 | 3.657 | 2-Methoxy-4-methylphenol | 93-51-6 | 13.53±1.66 | - | - | 1184 | 1193-S | MS, RI |
| 161 | 56.4095 | 1.256 | 1-Dodecene | 112-41-4 | 24.33±7.82 | 19.49±0.71 | 24.91±1.73 | 1192 | 1190-S | MS, RI |
| 162 | 56.5261 | 3.412 | 3,5-dimethyl-Phenol | 108-68-9 | 11.04±4.69 | - | - | 1193 | 1171-S | MS, RI |
| 163 | 58.2762 | 1.212 | (E)-3-Dodecene | 7206-14-6 | 12.55±2 | 8.71±0.22 | 10.3±1.05 | 1205 | 1185-S | MS, RI |
| 164 | 58.976 | 3.121 | 4,7-dimethyl-Benzofuran | 28715-26-6 | 6.26±1.52 | - | - | 1211 | 1220-S | MS, RI |
| 165 | 60.4925 | 4.822 | 2-Methyl-7-azaindole | 23612-48-8 | - | 5.82±0.35 | - | 1222 | - | MS, RI |
| 166 | 61.0761 | 1.282 | 2-butyl-1,1,3-trimethyl-Cyclohexane | 54676-39-0 | 4.72±0.77 | 3.73±0.03 | 3.88±0.45 | 1226 | 1228-S | MS, RI |
| 167 | 61.1927 | 3.217 | 3-ethyl-5-methyl-Phenol | 698-71-5 | 17.03±1.98 | - | 21.54±3.87 | 1227 | - | MS, RI |
| 168 | 61.5425 | 4.789 | Quinoline | 91-22-5 | - | 6.53±0.39 | - | 1230 | 1237-S | MS, RI |
| 169 | 61.8928 | 4.019 | 3-ethyl-4-methyl-pyrrole-2,5-dione | 20189-42-8 | 18.7±5.17 | - | - | 1232 | 1239-S | MS, RI |
| 170 | 62.0094 | 5.387 | Benzenepropanenitrile | 645-59-0 | 11.06±3.43 | 12.17±0.94 | 11.06±2.02 | 1233 | 1244-S | MS, RI |
| 171 | 62.7092 | 4.123 | Benzylacetone | 2550-26-7 | - | 3.03±0.61 | - | 1238 | 1232-S | MS, RI |
| 172 | 63.5259 | 3.181 | 2-Ethyl-1-H-indene | 17059-50-6 | - | 4.23±0.35 | 6.4±3.45 | 1244 | - | MS, RI |
| 173 | 64.6926 | 3.132 | 1,3-dimethyl-1H-Indene | 2177-48-2 | - | 9.14±0.88 | - | 1253 | - | MS, RI |
| 174 | 65.1591 | 1.263 | 2-Methyl-1-dodecene | 16435-49-7 | - | 1.04±0.05 | - | 1256 | 1285-S | MS, RI |
| 175 | 65.1589 | 2.223 | Hexylbenzene | 1077-16-3 | - | - | 3.74±0.75 | 1256 | 1247-S | MS, RI |
| 176 | 66.559 | 3.318 | 2,4,5-TriMethylphenol | 496-78-6 | - | 3.06±1.34 | - | 1267 | 1228-S | MS, RI |
| 177 | 66.9096 | 3.577 | 4-ethyl-2-methoxy-Phenol | 2785-89-9 | 14.44±6.23 | - | - | 1269 | 1282-S | MS, RI |
| 178 | 67.1429 | 5.207 | 1-Indanone | 83-33-0 | 17±4.12 | 10.67±0.16 | 8.81±1.45 | 1271 | 1289-S | MS, RI |
| 179 | 68.8923 | 1.444 | (Z)-6-Pentadecen-1-ol | 68797-95-5 | - | 0.96±0.05 | - | 1284 | - | MS, RI |
| 180 | 69.3596 | 4.117 | 2-Methyl-5,6,7,8-tetrahydroquinoxaline | 38917-65-6 | 3.09±1.4 | - | - | 1287 | - | MS, RI |
| 181 | 69.3596 | 5.449 | Indole | 120-72-9 | 61.13±8.25 | 51.88±1.22 | - | 1288 | 1295-S | MS, RI |
| 182 | 69.4762 | 3.614 | Bicyclo[4.4.1]undeca-1,3,5,7,9-pentaene | 2443-46-1 | 19.53±3.67 | - | - | 1288 | - | MS, RI |
| 183 | 70.0593 | 1.284 | 1-Tridecene | 2437-56-1 | 40.3±4.99 | 33.24±3.4 | 42.36±1.78 | 1292 | 1292-S | MS, RI |
| 184 | 70.8761 | 4.516 | 2-Methyl-1-indanone | 17496-14-9 | 5.8±1.66 | - | 3.31±0.93 | 1299 | - | MS, RI |
| 185 | 71.4596 | 3.888 | 1-Methylnaphthalene | 90-12-0 | 15.88±2.17 | - | 11.74±1.15 | 1303 | 1307-S | MS, RI |
| 186 | 71.9261 | 3.91 | 4-Hydroxy-3-methoxystyrene | 7786-61-0 | 23.63±3.18 | 9.26±1.85 | 9.02±1.81 | 1307 | 1317-S | MS, RI |
| 187 | 74.6096 | 1.492 | trans-4-Tridecen-1-yl acetate | 72269-48-8 | 5.8±1.13 | 3.33±0.3 | 3.92±1.04 | 1327 | - | MS, RI |
| 188 | 76.0095 | 3.548 | Nicotine | 54-11-5 | 412.77±59.84 | 288.89±44.02 | 417.09±25.24 | 1338 | 1361-S | MS, RI |
| 189 | 77.6427 | 2.802 | 1,2,3,4-tetrahydro-1,6,8-trimethyl-Naphthalene | 30316-36-0 | 12.12±2.19 | 10.16±1.34 | 9.89±1.37 | 1350 | - | MS, RI |
| 190 | 78.4595 | 2.448 | Solanone | 54868-48-3 | 44.13±4.86 | 23.16±0.6 | 22.3±1.16 | 1356 | - | MS, RI |
| 191 | 79.7427 | 3.024 | 1,1,3-trimethyl-1H-Indene | 2177-45-9 | 18.22±3.39 | 10.03±1.04 | 9.3±3.17 | 1366 | - | MS, RI |
| 192 | 80.4429 | 1.834 | α-copaene | 3856-25-5 | 7.29±1.05 | - | - | 1372 | 1376-S | MS, RI |
| 193 | 80.5591 | 3.979 | Biphenyl | 92-52-4 | - | 3.17±0.4 | - | 1373 | 1381-S | MS, RI |
| 194 | 81.1426 | 5.154 | 4-Methylindole | 16096-32-5 | 42.93±7.11 | 39.76±2.44 | 38.69±2.37 | 1377 | - | MS, RI |
| 195 | 83.1261 | 1.301 | 1-Tetradecene | 1120-36-1 | 21.18±2.69 | 18±1.11 | 21.91±0.57 | 1392 | 1392-S | MS, RI |
| 196 | 83.2427 | 1.891 | α-Guaiene | 3691-12-1 | 45.38±24.75 | - | - | 1393 | 1439-S | MS, RI |
| 197 | 83.8262 | 5.02 | 7-Methylindan-1-one | 39627-61-7 | 5.78±1.08 | - | - | 1398 | - | MS, RI |
| 198 | 84.7596 | 1.333 | 7-Tetradecene | 10374-74-0 | 1.94±0.4 | 1.62±0.28 | 2.45±0.26 | 1405 | 1369-S | MS, RI |
| 199 | 84.9922 | 2.047 | β-Elemene | 515-13-9 | - | - | 6.71±0.61 | 1407 | 1391-S | MS, RI |
| 200 | 85.8096 | 5.032 | Myosmine | 532-12-7 | 53.78±10.22 | 33.71±2.09 | 36.18±2.22 | 1414 | 1427-S | MS, RI |
| 201 | 85.8096 | 3.632 | 1,7-dimethyl-Naphthalene | 575-37-1 | 19.86±6.43 | 11.83±0.88 | 10.01±1.46 | 1414 | 1404-S | MS, RI |
| 202 | 86.743 | 2.015 | 2-Isopropenyl-4a,8-dimethyl-1,2,3,4,4a,5,6,8a-octahydronaphthalene | 207297-57-2 | 21.46±5.88 | - | - | 1421 | - | MS, RI |
| 203 | 88.3761 | 1.773 | 2,3,5,8-tetramethyl-1,5,9-Decatriene | 230646-72-7 | 6.21±2.17 | 10.09±0.76 | - | 1434 | - | MS, RI |
| 204 | 90.4762 | 1.909 | cis-β-Farnesene | 28973-97-9 | 28.96±5.51 | - | - | 1451 | 1444-S | MS, RI |
| 205 | 90.5928 | 2.167 | Amorphadiene | 92692-39-2 | 9.16±2.25 | - | - | 1452 | 1458-S | MS, RI |
| 206 | 90.709 | 4.751 | 3-Phenylpyridine | 1008-88-4 | - | 8.64±0.22 | 9.22±0.97 | 1453 | 1467-S | MS, RI |
| 207 | 90.9426 | 1.223 | 2-hexyl-1-Decanol | 2425-77-6 | - | 9.25±0.26 | 10.33±0.78 | 1455 | 1504-S | MS, RI |
| 208 | 92.8095 | 5.487 | Nicotyrine | 487-19-4 | 23.56±3.1 | 22.98±0.25 | - | 1470 | 1488-S | MS, RI |
| 209 | 92.9261 | 5.07 | 2,4,6-trimethyl-Benzonitrile | 2571-52-0 | 12.72±2.84 | 11.19±0.74 | 12.74±0.35 | 1471 | - | MS, RI |
| 210 | 93.7428 | 2.319 | α-curcumene | 644-30-4 | 10.55±6.43 | - | - | 1478 | 1483-S | MS, RI |
| 211 | 93.9761 | 5.014 | 2,3-Dimethylindole | 91-55-4 | 13±2.3 | 12.86±1.24 | 10.25±0.66 | 1480 | 1507-S | MS, RI |
| 212 | 96.7763 | 2.015 | α-Farnesene | 502-61-4 | 36.37±2.86 | 3.79±0.4 | 7.46±0.84 | 1502 | 1508-S | MS, RI |
| 213 | 96.8929 | 2.815 | 2,4-Di-tert-butylphenol | 96-76-4 | 7.13±1.18 | 5.22±0.44 | - | 1503 | 1519-S | MS, RI |
| 214 | 97.2422 | 4.969 | Anatabine | 2743-90-0 | - | - | 21.17±7.47 | 1507 | - | MS, RI |
| 215 | 97.476 | 2.058 | α-Bulnesene | 3691-11-0 | 6.42±1.61 | - | - | 1508 | 1505-S | MS, RI |
| 216 | 98.0595 | 2.352 | β-Cadinene | 523-47-7 | 3.09±1.52 | - | - | 1513 | 1518-S | MS, RI |
| 217 | 98.4094 | 4.846 | Dihydroactinidiolide | 17092-92-1 | 10.51±1.75 | 7.26±0.37 | 9.19±1.07 | 1517 | 1532-S | MS, RI |
| 218 | 98.7593 | 5.564 | 2,3'-Dipyridyl | 581-50-0 | 37.91±6.82 | 35.51±1.33 | 44.71±2.63 | 1520 | 1556-S | MS, RI |
| 219 | 98.9929 | 3.413 | 2,3,5-TriMethylnaphthalene | 2245-38-7 | 12.13±2.32 | - | - | 1522 | 1568-S | MS, RI |
| 220 | 104.8262 | 2.019 | (3E,7E)-4,8,12-Trimethyltrideca-1,3,7,11-tetraene | 62235-06-7 | 10.64±5.19 | - | - | 1572 | 1577-S | MS, RI |
| 221 | 107.2756 | 1.235 | Cetene | 629-73-2 | - | 9.73±0.37 | 10.97±1.01 | 1593 | 1592-S | MS, RI |
| 222 | 108.3261 | 3.548 | 3-Hydroxy-β-damascone | 102488-09-5 | 32.11±11.48 | - | 13.46±1.82 | 1603 | 1618-S | MS, RI |
| 223 | 115.9095 | 5.113 | Cotinine | 486-56-6 | 10.51±4.23 | - | - | 1689 | 1713-S | MS, RI |
| 224 | 126.176 | 0.927 | Neophytadiene | 504-96-1 | 97.08±20.87 | 74.2±13.91 | 116.84±16.5 | 1835 | 1837-S | MS, RI |
| 225 | 126.5263 | 1.123 | Phytone | 502-69-2 | 18.57±4.11 | 13.12±1.66 | 14.62±2.11 | 1840 | 1844-S | MS, RI |
| 226 | 126.6429 | 0.863 | 3,7,11,15-Tetramethylhexadec-2-ene | 2437-93-6 | 20.66±4.43 | 16.37±1.23 | 19.87±1.24 | 1842 | 1830-S | MS, RI |
| 227 | 128.8596 | 0.925 | Phytyl Acetate | 10236-16-5 | 19.45±5.07 | - | - | 1879 | - | MS, RI |
| 228 | 129.6757 | 0.871 | 1-Nonadecene | 18435-45-5 | - | - | 9.61±1.73 | 1892 | 1892-S | MS, RI |
| 229 | 130.6094 | 1.245 | β-Springene | 70901-63-2 | 27.63±12.9 | - | 9.31±0.99 | 1909 | - | MS, RI |
| 230 | 130.8425 | 1.178 | 2,6,10,14,18-Pentamethyl-2,6,10,14,18-eicosapentaene | 75581-03-2 | - | 13.73±3.49 | 26.89±1.8 | 1913 | - | MS, RI |
| 231 | 131.1928 | 1.552 | Cembrene | 1898-13-1 | 26.01±17.23 | 10.55±5.14 | 21.69±2.1 | 1920 | 1939-S | MS, RI |
| 232 | 131.4255 | 1.949 | 13-Heptadecyn-1-ol | 56554-77-9 | - | - | 5.58±0.41 | 1924 | - | MS, RI |
| 233 | 133.2929 | 1.268 | (E,E,E)-3,7,11,15-Tetramethylhexadeca-1,3,6,10,14-pentaene | 77898-97-6 | 26.41±2.91 | - | - | 1959 | 1994-S | MS, RI |
| 234 | 138.3094 | 1.691 | 4,8,13-Cyclotetradecatriene-1,3-diol, 1,5,9-trimethyl-12-(1-methylethyl)- | 7220-78-2 | 140.59±55.84 | 68.28±8.36 | 52.78±9.41 | 2056 | - | MS, RI |
| 235 | 140.7592 | 1.135 | Phytol | 150-86-7 | - | 8.66±0.47 | 12.63±4.97 | 2105 | 2114-S | MS, RI |
| "-" indicates that the compound was not detected. | | | | | | | | | | |

Table S2 ROAVs of VOCs in dfifferent cigar smoke.

| No. | Compound | Odor Thresholds (mg/m3) | ROAV | | |
| --- | --- | --- | --- | --- | --- |
|  |  |  | YX | DH | PE |
| 1 | Benzene | 15 | 0.0003 | - | 0.0007 |
| 2 | 3-Penten-2-one | ND | - | - | - |
| 3 | Cyclohexene | 0.6 | - | - | 0.0081 |
| 4 | 1-Heptene | 1.5 | - | 0.0054 | 0.0068 |
| 5 | 2,4-Dimethylfuran | ND | - | - | - |
| 6 | Methyl Isobutyl Ketone | 1.9 | - | - | 0.0060 |
| 7 | Pyridine | 0.078 | - | 0.2506 | 0.6488 |
| 8 | Disulfide, dimethyl | 0.029 | - | 0.2437 | 0.2294 |
| 9 | (1-methylethylidene)-Cyclobutane | ND | - | - | - |
| 10 | cis-1,2,trans-1,3-1,2,3-Trimethylcyclopentane in cyclohexane | ND | - | - | - |
| 11 | 2,3-dimethyl-1,3-Pentadiene | ND | - | - | - |
| 12 | Toluene | 2 | 0.0007 | - | - |
| 13 | 1-methyl-Cyclohexene | ND | - | - | - |
| 14 | 6-methyl-2-Heptene | ND | - | - | - |
| 15 | 1-methyl-1,4-Cyclohexadiene | ND | - | - | - |
| 16 | 3-Hexanone | ND | - | - | - |
| 17 | 2-methyl-1-Heptene | ND | - | - | - |
| 18 | 2-Hexanone | 0.28 | 0.0009 | - | 0.0206 |
| 19 | 1-Octene | 0.33 | - | - | 0.0479 |
| 20 | Cyclopentanone | 31 | - | 0.0008 | 0.0008 |
| 21 | 1-Ethylpyrrole | ND | - | - | - |
| 22 | 2-methyl-Pyridine | 0.05 | 0.0186 | 0.4669 | - |
| 23 | 2-Methylpyrazine | 1.9 | - | 0.0080 | - |
| 24 | Furfural | 0.008 | - | 0.1275 | - |
| 25 | (E)-2-Hexenal | 0.034 | 0.0650 | 1.0715 | - |
| 26 | 3-Methylpyrrole | ND | - | - | - |
| 27 | 2,6-dimethyl-3-Heptene | ND | - | - | - |
| 28 | 2,6-dimethyl-1-Heptene | ND | - | - | - |
| 29 | 3-Methylcyclopentanone | ND | - | - | - |
| 30 | Furfurylalkohol | 32 | 0.0000 | 0.0001 | - |
| 31 | Lurasidone | ND | - | - | - |
| 32 | 1,2,4,4-Tetramethylcyclopentene | ND | - | - | - |
| 33 | 3-Picoline | ND | - | - | - |
| 34 | Ethylbenzene | 2 | 0.0007 | 0.0132 | 0.0165 |
| 35 | (E,E)-2,4-Hexadienal | 0.0018 | - | 6.3566 | 7.2218 |
| 36 | m-Xylene | 0.6 | - | 0.0884 | 0.0807 |
| 37 | Phenylethyne | ND | - | - | - |
| 38 | 2,6-Lutidine | 0.003 | 0.2107 | 3.9222 | 2.5149 |
| 39 | 1-Nonene | 0.0028 | 0.0928 | - | - |
| 40 | p-Xylene | 0.6 | - | - | 0.0260 |
| 41 | 1,3,5,7-Cyclooctatetraene | ND | - | - | - |
| 42 | Cyclohexanone | 0.48 | 0.0004 | - | - |
| 43 | 2-ethyl-Pyridine | 0.057 | 0.0119 | 0.1865 | 0.1645 |
| 44 | 2-Cyclopenten-1-one, 2-methyl- | ND | - | - | - |
| 45 | 3,3,6-trimethyl-1,5-Heptadiene | ND | - | - | - |
| 46 | Ethanone, 1-(2-furanyl)- | ND | - | - | - |
| 47 | 3,7-dimethyl-1-Octene | ND | - | - | - |
| 48 | 2,5-dimethyl-Pyrazine | 0.17 | 0.0059 | 0.0743 | 0.0713 |
| 49 | 2-Ethylpyrazine | 0.25 | 0.0027 | 0.0334 | 0.0376 |
| 50 | 1,5,5-trimethyl-3-methylene-Cyclohexene | ND | - | - | - |
| 51 | Cumene | 0.025 | - | 0.0740 | - |
| 52 | 2,4-dimethyl-Pyridine | ND | - | - | - |
| 53 | 2,5-dimethyl-Pyridine | ND | - | - | - |
| 54 | 2-methyl-3-Heptanone | ND | - | - | - |
| 55 | 2-ethyl-Cyclopentanone | ND | - | - | - |
| 56 | β-Citronellene | ND | - | - | - |
| 57 | Allylbenzene | ND | - | - | - |
| 58 | (1α,3α,6α)-3,7,7-trimethyl-Bicyclo[4.1.0]heptane | ND | - | - | - |
| 59 | 3-methyl-Pentanoic acid | ND | - | - | - |
| 60 | 1-Phenylpropane | 0.019 | 0.0427 | - | 0.5764 |
| 61 | 3-ethyl-Pyridine | ND | - | - | - |
| 62 | 1-ethyl-3-methyl-Benzene | 0.088 | 0.0186 | 0.1716 | - |
| 63 | 2-ethyl-6-methyl-Pyridine | ND | - | - | - |
| 64 | 1-ethyl-2-methyl-Benzene | 0.36 | - | - | 0.0467 |
| 65 | 3-methyl-2-Cyclopenten-1-one | ND | - | - | - |
| 66 | Benzaldehyde | 0.014 | 0.1004 | - | - |
| 67 | 1-ethyl-4-methyl-Benzene | 0.041 | - | - | 0.2360 |
| 68 | Mesitylene | 0.18 | - | 0.0277 | - |
| 69 | 2-methyl-6-methylene-1,7-Octadiene | ND | - | - | - |
| 70 | α-Methylstyrene | 0.1 | 0.0044 | 0.0459 | 0.0526 |
| 71 | 1,5,5,6-tetramethyl-1,3-Cyclohexadiene | ND | - | - | - |
| 72 | Benzonitrile | ND | - | - | - |
| 73 | 2,6-dimethyl-2-trans-6-octadiene | ND | - | - | - |
| 74 | 6-methyl-5-Hepten-2-one | 0.0063 | 0.1220 | 1.2173 | - |
| 75 | 3,4-dimethylcyclopent-2-en-1-one | ND | - | - | - |
| 76 | 2,6-dimethyl-3,5-Heptadien-2-ol | ND | - | - | - |
| 77 | 1-Decene | 37 | 0.0000 | 0.0003 | 0.0003 |
| 78 | 2-Methylstyrene | ND | - | - | - |
| 79 | 2-ethyl-6-methyl-Pyrazine | 0.04 | 0.0193 | 0.3160 | 0.3115 |
| 80 | 2-ethyl-3-methyl-Pyrazine | 0.035 | 0.0315 | 0.5328 | 0.4961 |
| 81 | 2,4,6-Collidine | ND | - | - | - |
| 82 | 2-ethyl-4-methyl-1H-Pyrrole | ND | - | - | - |
| 83 | 3-Pyridinecarbonitrile | ND | - | - | - |
| 84 | 3-Carene | 9.1 | 0.0001 | 0.0005 | - |
| 85 | 2,5,6-Trimethyl-1,3,6-heptatriene | ND | - | - | - |
| 86 | 4-methyl-1-Decene | ND | - | - | - |
| 87 | α-Terpinene | 2.35 | 0.0003 | - | - |
| 88 | 2-Carene | ND | - | - | - |
| 89 | o-Cymene | 0.004 | - | - | 0.3077 |
| 90 | 1,2,4-Trimethylbenzene | 0.14 | 0.0124 | - | - |
| 91 | 3-methyl-1,2-Cyclopentanedione | 0.01 | 0.1973 | - | - |
| 92 | p-Menth-1-ene | ND | - | - | - |
| 93 | 1,3,5-tris(methylene)-Cycloheptane | ND | - | - | - |
| 94 | D-Limonene | 0.0059 | 1.4813 | - | 20.1523 |
| 95 | 2,3-dimethyl-2-Cyclopenten-1-one | ND | - | - | - |
| 96 | N-Methyl-2-pyrrolidone | 17.113 | - | 0.0038 | 0.0052 |
| 97 | 1,2,3-Trimethylbenzene | ND | - | - | - |
| 98 | 2,7,10-trimethyl-Dodecane | ND | - | - | - |
| 99 | 3,8-p-Menthadiene | ND | - | - | - |
| 100 | 3-phenyl-1-Propyne | ND | - | - | - |
| 101 | 4-Phenyl-1-butene | ND | - | - | - |
| 102 | 1,3-diethyl-Benzene | 0.39 | 0.0012 | 0.0060 | 0.0065 |
| 103 | 3-Propyltoluene | ND | - | - | - |
| 104 | o-Cresol | 0.0004 | 4.2128 | 79.6612 | 81.8907 |
| 105 | Butylbenzene | 0.014 | 0.1135 | 1.1008 | 1.1924 |
| 106 | 5-methyldecane | ND | - | - | - |
| 107 | 2-Acetyl pyrrole | 2 | 0.0001 | 0.0011 | - |
| 108 | 2-Propyltoluene | ND | - | - | - |
| 109 | 2-ethyl-4,6-dimethyl-Pyridine | ND | - | - | - |
| 110 | 3-ethyl-2-Cyclopenten-1-one | ND | - | - | - |
| 111 | Tetracyclo[5.3.0.0<2,6>.0<3,10>]deca-4,8-diene | ND | - | - | - |
| 112 | 2-Methylindene | ND | - | - | - |
| 113 | m,α-dimethylstyrene | ND | - | - | - |
| 114 | 6-isopropylidene-1-methyl-Bicyclo[3.1.0]hexane | ND | - | - | - |
| 115 | Guaiacol | 0.0001 | 14.7846 | 92.7913 | 100.0000 |
| 116 | 2,3,4-trimethylcyclopent-2-en-1-one | ND | - | - | - |
| 117 | 2-ethyl-3,5-dimethyl-Pyrazine | 0.00001 | 16.4624 | - | - |
| 118 | Terpinolene | ND | - | - | - |
| 119 | 2-Ethyl-p-xylene | ND | - | - | - |
| 120 | 5-Ethyl-m-xylene | ND | - | - | - |
| 121 | o-Tolunitrile | ND | - | - | - |
| 122 | 4-Isopropenyltoluene | 0.665 | 0.0027 | - | - |
| 123 | 1-Undecene | ND | - | - | - |
| 124 | 4-Ethenyl-1,2-dimethylbenzene | ND | - | - | - |
| 125 | 2,6-dimethyl-Phenol | 0.0002 | 7.8615 | 100.0000 | 83.7096 |
| 126 | 1-methyl-4-(1-methylpropyl)-Benzene | ND | - | - | - |
| 127 | Phenethyl alcohol | 0.01 | 0.1010 | - | - |
| 128 | 1,3,8-p-Menthatriene | 0.015 | 0.0876 | 0.4134 | 0.4706 |
| 129 | Ethylcyclopentenolone | ND | - | - | - |
| 130 | (E)-4,8-Dimethylnona-1,3,7-triene | ND | - | - | - |
| 131 | 1-pentyl-2-propyl-Cyclopropane | ND | - | - | - |
| 132 | 3-(1-methylethenyl)-Cyclooctene | ND | - | - | - |
| 133 | Isophorone | 0.0017 | 0.1297 | 1.4642 | - |
| 134 | 2-Methyl-1-phenylpropene | ND | - | - | - |
| 135 | 2-ethenyl-1,1-dimethyl-3-methylene-Cyclohexane | ND | - | - | - |
| 136 | 1-methyl-3-(1-methyl-2-propenyl)-Benzene | ND | - | - | - |
| 137 | Neo-alloocimene | ND | - | - | - |
| 138 | (3E,5E)-2,6-Dimethyl-1,3,5,7-octatetrene | ND | - | - | - |
| 139 | Benzeneacetonitrile | ND | - | - | - |
| 140 | (3-methyl-3-butenyl)-Benzene | ND | - | - | - |
| 141 | 2-Ethylphenol | ND | - | - | - |
| 142 | 1-ethyl-4-(1-methylethyl)-Benzene | ND | - | - | - |
| 143 | 1,2-Dihydronaphthalene | ND | - | - | - |
| 144 | 1-Allyl-2-methylbenzene | ND | - | - | - |
| 145 | 2-Isobutyl-4-methylpyridine | ND | - | - | - |
| 146 | 1,2,3,4-Tetramethylbenzene | 0.061 | - | - | 0.1039 |
| 147 | 6-methylundecane | ND | - | - | - |
| 148 | Phenylpentane | 6 | 0.0001 | 0.0010 | 0.0011 |
| 149 | 6-Ethyl-5,6-dihydro-2H-pyran-2-one | ND | - | - | - |
| 150 | 1-methyl-4-butylBenzene | ND | - | - | - |
| 151 | 5-propyl-Decane | ND | - | - | - |
| 152 | 4-Ethylphenol | ND | - | - | - |
| 153 | 3-ethyl-Phenol | 0.001 | 1.8001 | - | - |
| 154 | 3-Methylundecane | ND | - | - | - |
| 155 | 2,3-dimethyl-1H-Indene | ND | - | - | - |
| 156 | 1-Furfurylpyrrole | ND | - | - | - |
| 157 | 2,4,6-Trimethylstyrene | ND | - | - | - |
| 158 | Naphthalene | 0.007 | 0.3057 | 4.7783 | 4.5513 |
| 159 | 2,2-dimethyl-1,3-dihydroindene | ND | - | - | - |
| 160 | 2-Methoxy-4-methylphenol | ND | - | - | - |
| 161 | 1-Dodecene | ND | - | - | - |
| 162 | 3,5-dimethyl-Phenol | 0.00004 | 19.1178 | - | - |
| 163 | (E)-3-Dodecene | ND | - | - | - |
| 164 | 4,7-dimethyl-Benzofuran | ND | - | - | - |
| 165 | 2-Methyl-7-azaindole | ND | - | - | - |
| 166 | 2-butyl-1,1,3-trimethyl-Cyclohexane | ND | - | - | - |
| 167 | 3-ethyl-5-methyl-Phenol | ND | - | - | - |
| 168 | Quinoline | 0.03 | - | 0.2813 | - |
| 169 | 3-ethyl-4-methyl-pyrrole-2,5-dione | ND | - | - | - |
| 170 | Benzenepropanenitrile | ND | - | - | - |
| 171 | Benzylacetone | ND | - | - | - |
| 172 | 2-Ethyl-1-H-indene | ND | - | - | - |
| 173 | 1,3-dimethyl-1H-Indene | ND | - | - | - |
| 174 | 2-Methyl-1-dodecene | ND | - | - | - |
| 175 | Hexylbenzene | 0.029 | - | - | 0.1638 |
| 176 | 2,4,5-TriMethylphenol | ND | - | - | - |
| 177 | 4-ethyl-2-methoxy-Phenol | 0.00001 | 100.0000 | - | - |
| 178 | 1-Indanone | ND | - | - | - |
| 179 | (Z)-6-Pentadecen-1-ol | ND | - | - | - |
| 180 | 2-Methyl-5,6,7,8-tetrahydroquinoxaline | ND | - | - | - |
| 181 | Indole | ND | - | - | - |
| 182 | Bicyclo[4.4.1]undeca-1,3,5,7,9-pentaene | ND | - | - | - |
| 183 | 1-Tridecene | ND | - | - | - |
| 184 | 2-Methyl-1-indanone | ND | - | - | - |
| 185 | 1-Methylnaphthalene | ND | - | - | - |
| 186 | 4-Hydroxy-3-methoxystyrene | 0.0004 | 4.0916 | 29.9077 | 28.5988 |
| 187 | trans-4-Tridecen-1-yl acetate | ND | - | - | - |
| 188 | Nicotine | 0.066 | 0.4332 | 5.6561 | 8.0188 |
| 189 | 1,2,3,4-tetrahydro-1,6,8-trimethyl-Naphthalene | ND | - | - | - |
| 190 | Solanone | ND | - | - | - |
| 191 | 1,1,3-trimethyl-1H-Indene | ND | - | - | - |
| 192 | α-copaene | ND | - | - | - |
| 193 | Biphenyl | 0.0033 | - | 1.2393 | - |
| 194 | 4-Methylindole | ND | - | - | - |
| 195 | 1-Tetradecene | ND | - | - | - |
| 196 | α-Guaiene | ND | - | - | - |
| 197 | 7-Methylindan-1-one | ND | - | - | - |
| 198 | 7-Tetradecene | ND | - | - | - |
| 199 | β-Elemene | ND | - | - | - |
| 200 | Myosmine | ND | - | - | - |
| 201 | 1,7-dimethyl-Naphthalene | ND | - | - | - |
| 202 | 2-Isopropenyl-4a,8-dimethyl-1,2,3,4,4a,5,6,8a-octahydronaphthalene | ND | - | - | - |
| 203 | 2,3,5,8-tetramethyl-1,5,9-Decatriene | ND | - | - | - |
| 204 | cis-β-Farnesene | ND | - | - | - |
| 205 | Amorphadiene | ND | - | - | - |
| 206 | 3-Phenylpyridine | ND | - | - | - |
| 207 | 2-hexyl-1-Decanol | ND | - | - | - |
| 208 | Nicotyrine | ND | - | - | - |
| 209 | 2,4,6-trimethyl-Benzonitrile | ND | - | - | - |
| 210 | α-curcumene | ND | - | - | - |
| 211 | 2,3-Dimethylindole | ND | - | - | - |
| 212 | α-Farnesene | ND | - | - | - |
| 213 | 2,4-Di-tert-butylphenol | ND | - | - | - |
| 214 | Anatabine | ND | - | - | - |
| 215 | α-Bulnesene | ND | - | - | - |
| 216 | β-Cadinene | ND | - | - | - |
| 217 | Dihydroactinidiolide | ND | - | - | - |
| 218 | 2,3'-Dipyridyl | ND | - | - | - |
| 219 | 2,3,5-TriMethylnaphthalene | ND | - | - | - |
| 220 | (3E,7E)-4,8,12-Trimethyltrideca-1,3,7,11-tetraene | ND | - | - | - |
| 221 | Cetene | ND | - | - | - |
| 222 | 3-Hydroxy-β-damascone | ND | - | - | - |
| 223 | Cotinine | ND | - | - | - |
| 224 | Neophytadiene | ND | - | - | - |
| 225 | Phytone | ND | - | - | - |
| 226 | 3,7,11,15-Tetramethylhexadec-2-ene | ND | - | - | - |
| 227 | Phytyl Acetate | ND | - | - | - |
| 228 | 1-Nonadecene | ND | - | - | - |
| 229 | β-Springene | ND | - | - | - |
| 230 | 2,6,10,14,18-Pentamethyl-2,6,10,14,18-eicosapentaene | ND | - | - | - |
| 231 | Cembrene | ND | - | - | - |
| 232 | 13-Heptadecyn-1-ol | ND | - | - | - |
| 233 | (E,E,E)-3,7,11,15-Tetramethylhexadeca-1,3,6,10,14-pentaene | ND | - | - | - |
| 234 | 4,8,13-Cyclotetradecatriene-1,3-diol, 1,5,9-trimethyl-12-(1-methylethyl)- | ND | - | - | - |
| 235 | Phytol | ND | - | - | - |
| "ND" indicates that the threshold was not queried and the OAV could not be calculated.  "-" indicates that the compound was not detected in the sample or the OAV could not be calculated because of the absent of threshold. | | | | | |
